# Supplementary material for: Ubiquitin Linkage-Specific Affimers Reveal Insights into K6-Linked Ubiquitin Signaling
Source: Mol Cell. 2017 Oct 5;68(1):233–246.e5. doi: 10.1016/j.molcel.2017.08.020 (PMC5640506; doi:10.1016/j.molcel.2017.08.020)
Supplement: Document S1. Figures S1–S7 [file mmc1.pdf]

Molecular Cell, Volume *68*

Supplemental Information

Ubiquitin Linkage-Specific Affimers Reveal Insights into K6-Linked Ubiquitin Signaling

Martin A. Michel, Kirby N. Swatek, Manuela K. Hospenthal, and David Komander

Figure S1

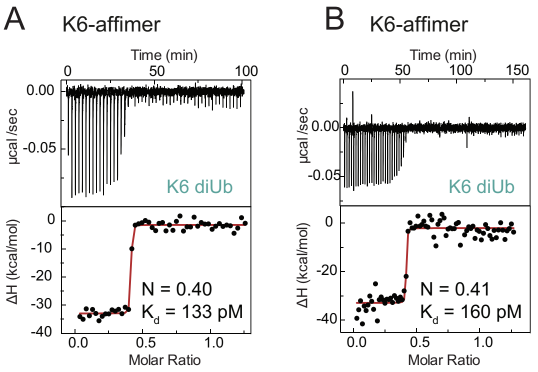

C

	K_d ± SEM (pM)	N ± SEM	ΔH ± SEM (kcal/mol)	ΔG (kcal/mol)
K33-affimer : K33 diUb	211 ± 280	0.44 ± 0.02	-16.2 ± 0.24	-24.6
K33-affimer : K11 diUb	7040 ± 4410	0.40 ± 0.07	-17.6 ± 0.70	-11.1
K6-affimer : K6 diUb	172 ± 579	0.46 ± 0.01	-33.3 ± 0.46	-13.3
K6-affimer : K6 diUb	133 ± 140	0.40 ± 0.02	-31.5 ± 0.46	-13.5
K6-affimer: K6 diUb	160 ± 233	0.41 ± 0.02	-30.8 ± 0.83	-13.4

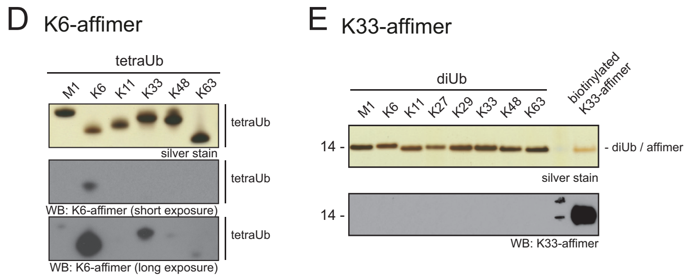

Supplementary Figure Legends

Figure S1 (related to Figure 1): Linkage-specificity of affimers

A-B) ITC curves of K6-affimer (5 µM, in cell) binding to K6 diUb (30 µM, in syringe) with A) 49 x 0.5 µL and B) 79 x 0.5 µL injections, respectively. Plots for raw heat (*top*) and derived isotherms (*bottom*) with fits (red) are shown. **C)** Table for best-fit parameters for ITC experiments from Figure 1 and S1. **D)** Western blot of the K6-affimer against differently linked tetraUb chains. **E)** Western blot with the biotinylated K33-affimer against differently linked diUb. Biotinylated K33-affimer was also loaded onto the gel as a technical control for detection with Streptavidin-HRP.

Figure S2

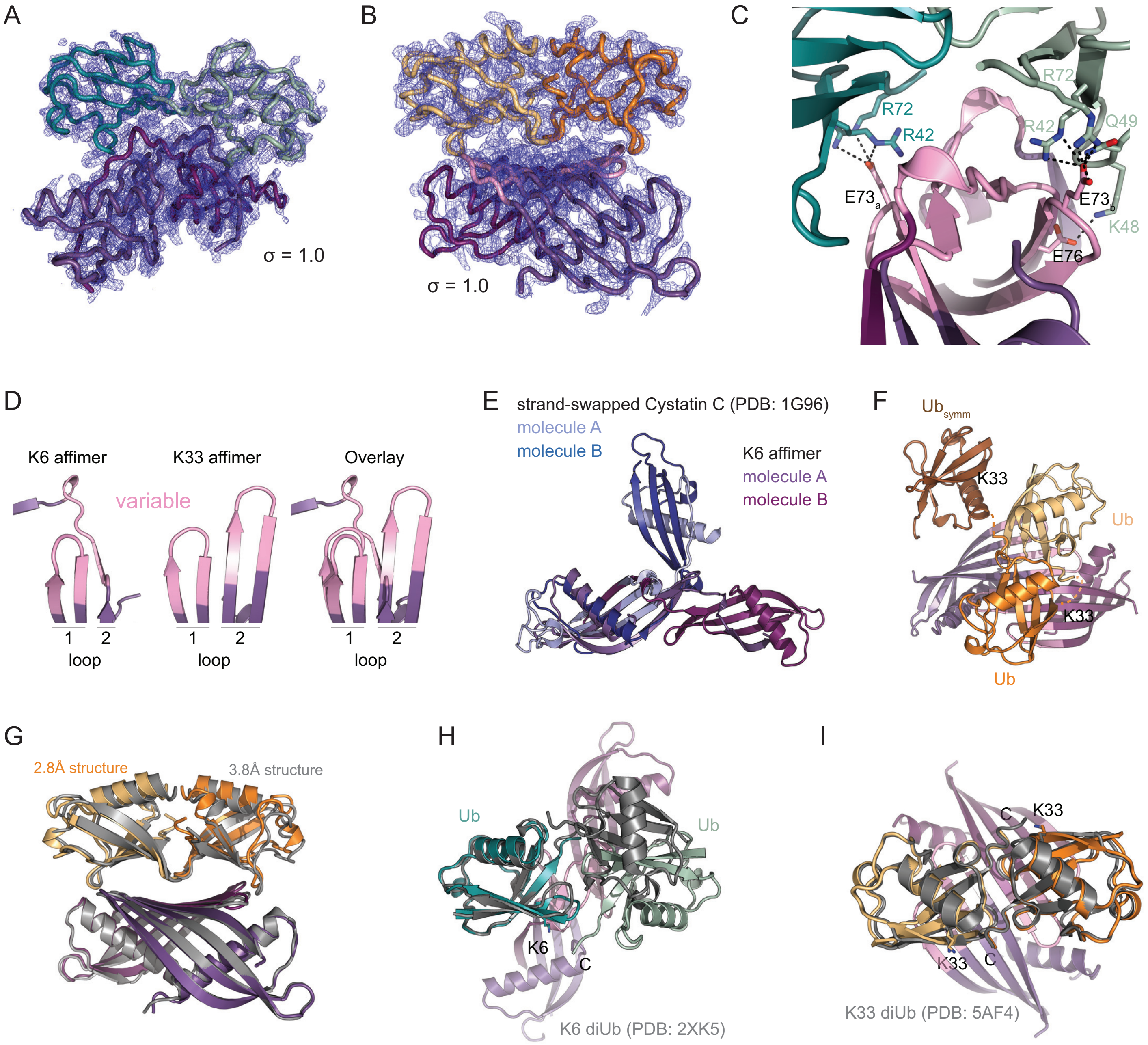

A
σ = 1.0
B
σ = 1.0
C
R72
R42
E73a
R72
R42
Q49
E73b
K48
E76
D
K6 affimer
K33 affimer
Overlay
variable
1 2
loop
1 2
loop
1 2
loop
E
strand-swapped Cystatin C (PDB: 1G96)
molecule A
molecule B
K6 affimer
molecule A
molecule B
F
Ubsymm
K33
Ub
K33
Ub
G
2.8Å structure
3.8Å structure
H
Ub
Ub
K6
C
K6 diUb (PDB: 2XK5)
I
C
K33
K33
C
K33 diUb (PDB: 5AF4)

Figure S2 (related to Figure 2): Structures of linkage-specific affimers with their cognate diUbs

A) Structure of K6-affimer bound to K6 diUb with corresponding 2 $|F_o|$ - $|F_c|$ electron density map, contoured at σ = 1.0. **B)** As in A, but for the K33-affimer:K33 diUb structure. **C)** Close up of charged interaction in the K6-affimer:K6 diUb structure, mediated by the variable region (pink) of the affimer. **D)** Variable loops (pink) of K6- and K33-affimers, and in overlay, showing the difference in secondary structure. **E)** Overlay of the strand-swapped K6-affimer (orange) with naturally occuring human cystatin C (blue, PDB: 1G96) that is also strand-swapped. Other naturally occuring cystatins engage in the strand swap in the same fashion as cystatin C, but the relative orientation of the two monomers differs. **F)** Symmetry-related molecules of the K33-affimer:K33 diUb structure showing how the bound chain can be extended. **G)** Overlay of the structure of the K33-affimer:K33 diUb in the *H3* space group (2.5Å) and the $P2_1$ space group (3.8Å, grey). **H)** Overlay of the K6-affimer:K6 diUb structure with previously solved K6 diUb structure (grey, PDB: 2XEW), superimposed on the distal Ub. **I)** As in E, but for the K33-affimer:K33 diUb complex and K33 diUb (grey, PDB: 5AF4), respectively.

Figure S3

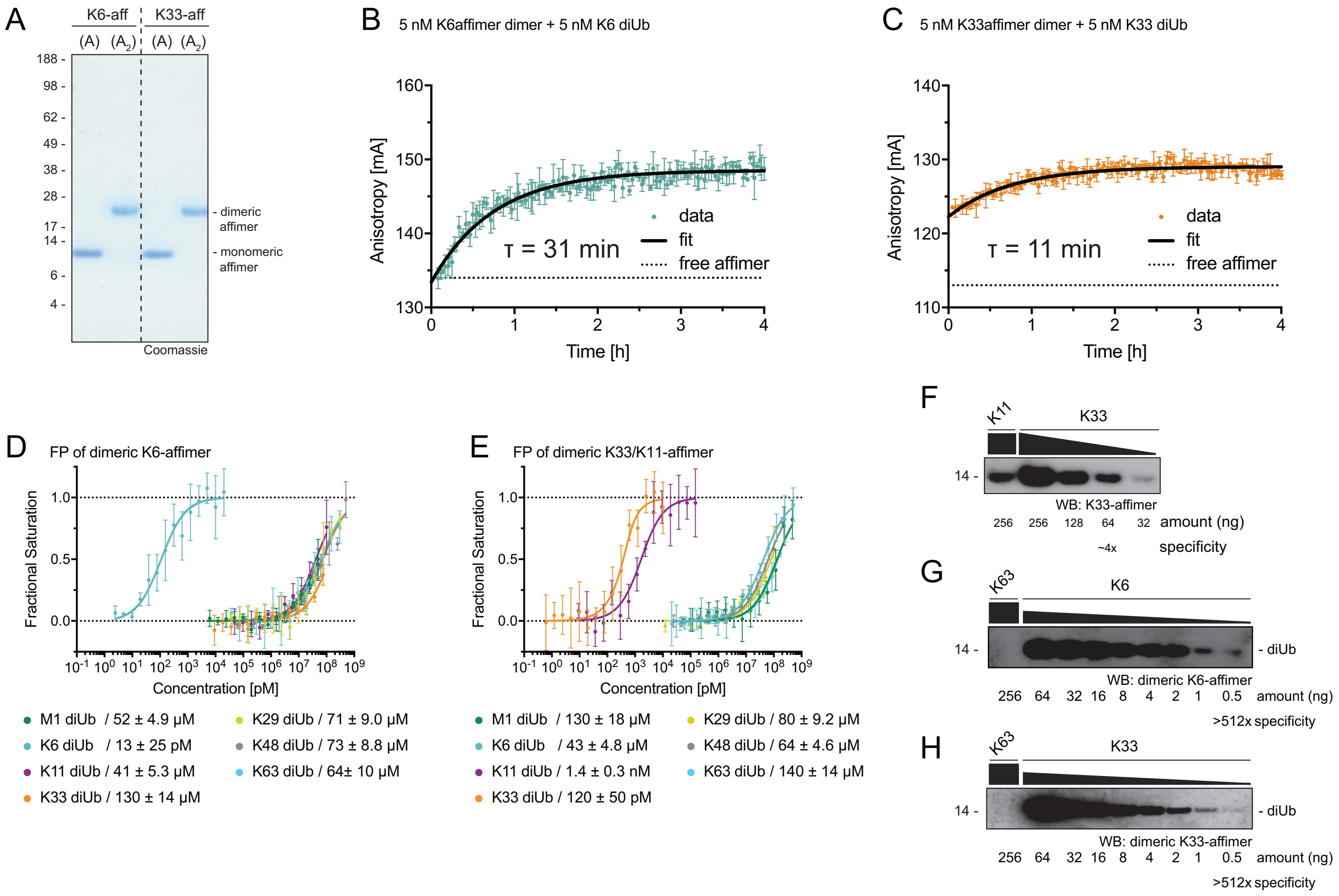

A
K6-aff
K33-aff
(A) (A_2) (A) (A_2)
188 -
98 -
62 -
49 -
38 -
28 -
17 -
14 -
6 -
4 -
- dimeric affimer
- monomeric affimer
Coomassie
B
5 nM K6affimer dimer + 5 nM K6 diUb
Anisotropy [mA]
Time [h]
τ = 31 min
data
fit
free affimer
C
5 nM K33affimer dimer + 5 nM K33 diUb
Anisotropy [mA]
Time [h]
τ = 11 min
data
fit
free affimer
D
FP of dimeric K6-affimer
Fractional Saturation
Concentration [pM]
M1 diUb / 52 ± 4.9 μM
K6 diUb / 13 ± 25 pM
K11 diUb / 41 ± 5.3 μM
K33 diUb / 130 ± 14 μM
K29 diUb / 71 ± 9.0 μM
K48 diUb / 73 ± 8.8 μM
K63 diUb / 64± 10 μM
E
FP of dimeric K33/K11-affimer
Fractional Saturation
Concentration [pM]
M1 diUb / 130 ± 18 μM
K6 diUb / 43 ± 4.8 μM
K11 diUb / 1.4 ± 0.3 nM
K33 diUb / 120 ± 50 pM
K29 diUb / 80 ± 9.2 μM
K48 diUb / 64 ± 4.6 μM
K63 diUb / 140 ± 14 μM
F
K11
K33
14 -
WB: K33-affimer
256 256 128 64 32 amount (ng)
~4x specificity
G
K63
K6
14 -
- diUb
WB: dimeric K6-affimer
256 64 32 16 8 4 2 1 0.5 amount (ng)
>512x specificity
H
K63
K33
14 -
- diUb
WB: dimeric K33-affimer
256 64 32 16 8 4 2 1 0.5 amount (ng)
>512x specificity

Figure S3 (related to Figure 3): Affimers dimerize to achieve linkage-specificity

A) Coomassie-stained gel of monomeric and dimerized versions of K6- and K33/K11-affimers, respectively. **B)** Fluorescence anisotropy time course with 5 nM K6-affimer and 5 nM K6 diUb. The dotted line indicates the anisotropy value for free K6-affimer and the data was fitted using a simple one-phase association model. **C)** As in B but for the K33/K11-affimer. **D)** Fluorescence polarization (FP) binding assay for the K6-affimer binding to differently linked diUb fitted with a one-site binding model accounting for ligand depletion. **E)** As in D, but for the K33/K11-affimer. **F)** Western blot using the dimerized K6-affimer using the indicated amounts of K6 diUb or K63 diUb .**G)** As in F, but probed with the dimerized K33/K11-affimer. **H)** Indicated amounts of K33 and K11 diUb were probed with the K33/K11-affimer by Western blotting. The specificity was estimated from relative signal strength and corresponding amounts loaded.

Figure S4

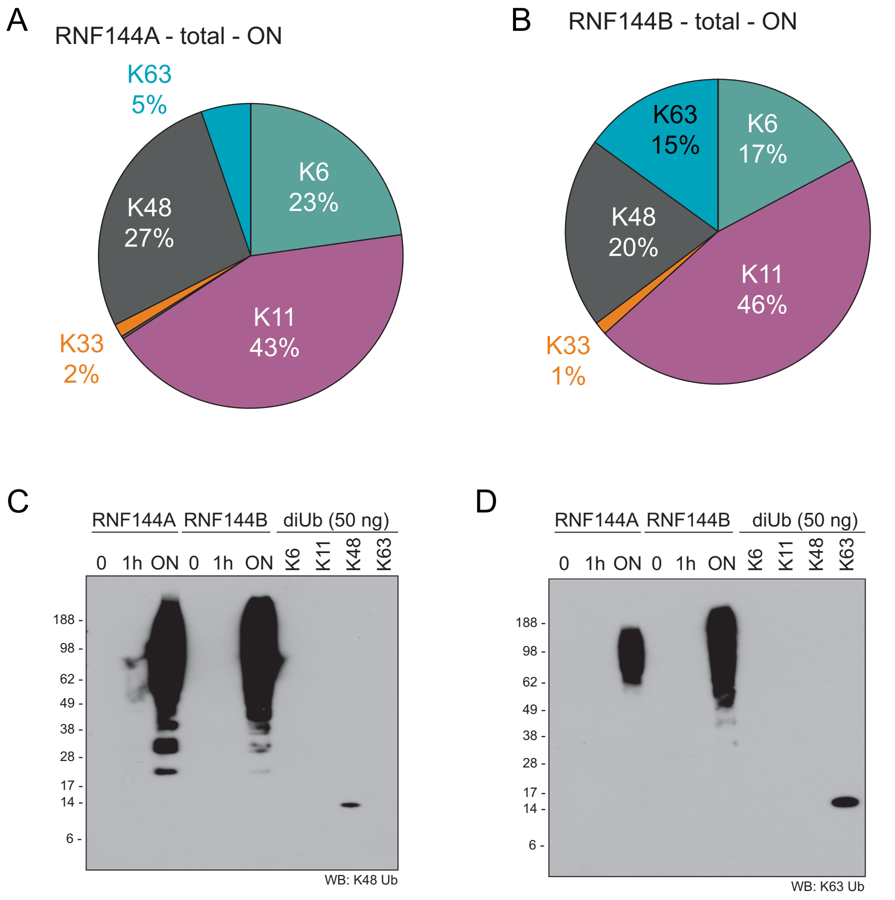
A
RNF144A - total - ON
K63
5%
K6
23%
K48
27%
K11
43%
K33
2%
B
RNF144B - total - ON
K63
15%
K6
17%
K48
20%
K11
46%
K33
1%
C
RNF144A
RNF144B
diUb (50 ng)
0
1h
ON
0
1h
ON
K6
K11
K48
K63
188 -
98 -
62 -
49 -
38 -
28 -
17 -
14 -
6 -
WB: K48 Ub
D
RNF144A
RNF144B
diUb (50 ng)
0
1h
ON
0
1h
ON
K6
K11
K48
K63
188 -
98 -
62 -
49 -
38 -
28 -
17 -
14 -
6 -
WB: K63 Ub

Figure S4 (related to Figure 4): *In vitro* applications of Affimers

A-B) AQUA-MS-derived Ub linkage composition for total assembly reaction of A) RNF144A and B) RNF144B after overnight assembly with UBE2L3 and Ub wt. **C-D)** *In vitro* assembly reaction of RNF144A and RNF144B with indicated amounts of recombinant diUb as in Figure 4D and probed with C) a K48-specific antibody or D) a K63-specific antibody. Longer chains are preferentially detected, probably due to avidity effects.

Figure S5

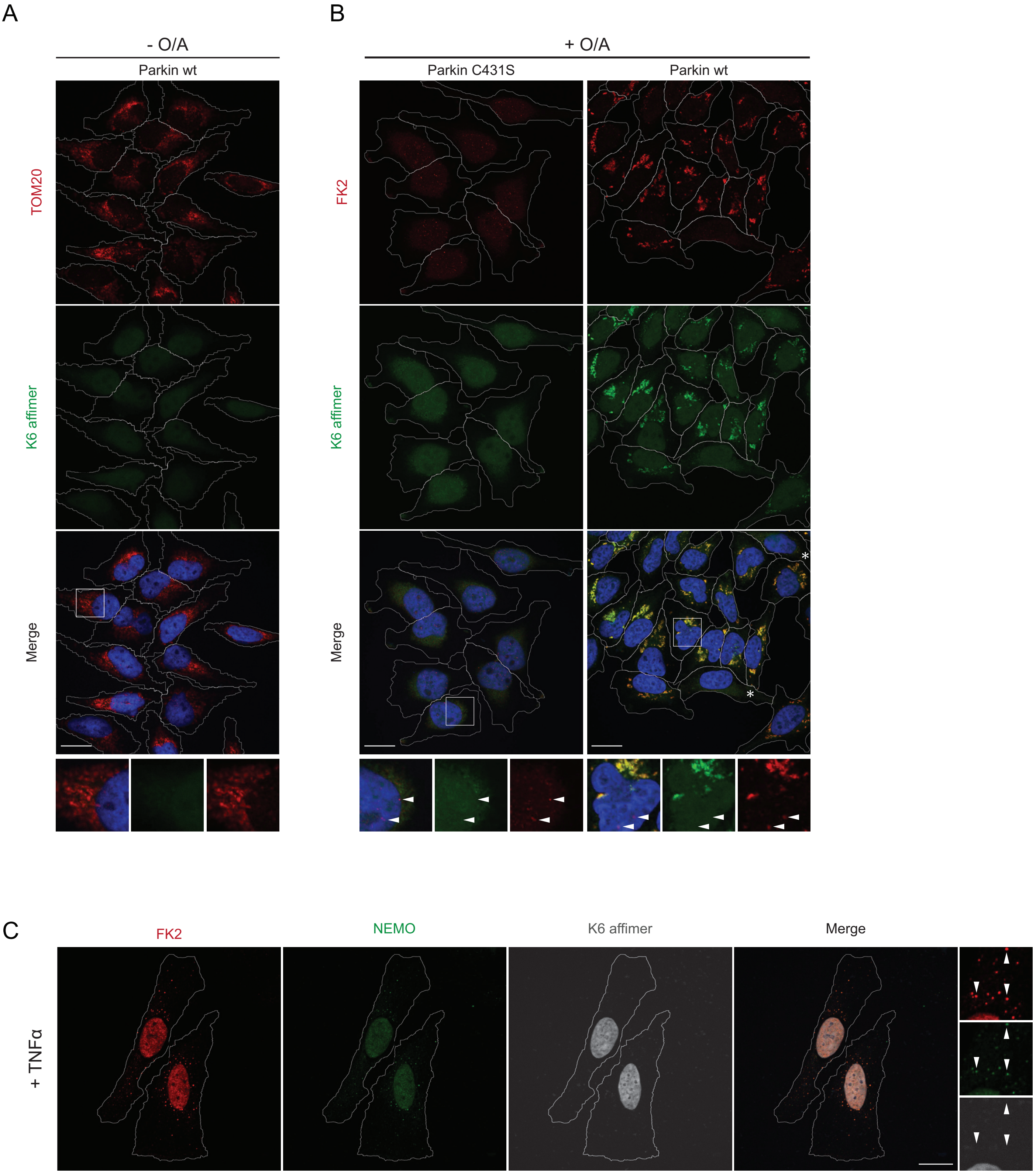
A
B
- O/A
+ O/A
Parkin wt
Parkin C431S
Parkin wt
TOM20
FK2
K6 affimer
K6 affimer
Merge
Merge
*
*
C
FK2
NEMO
K6 affimer
Merge
+ TNFα

Figure S5 (related to Figure 5): *In vivo* applications of Affimers

A) Confocal fluorescence microscopy images as in Figure 5C of untreated HeLa cells expressing wt Parkin stained with TOM20 (red), K6-affimer (green) and DAPI (blue). Scale bars correspond to 20 μm. **B)** As in A, but cells were treated with O/A and either wt Parkin or catalytically inactive (C431S) Parkin was expressed. Cells were stained with FK2 (red), K6-affimer (green) and DAPI (blue). Arrows indicate FK2-labelled ubiquitin conjugates that are not recognized by the K6-affimer. Not all cells seem to have ubiquitinated mitochondria (asterisks), either due to loss of Parkin expression, or because mitophagy was not induced. **C)** Confocal fluorescence microscopy images of cells that were treated with TNFα and stained with FK2 (red), NEMO (green), K6-affimer (grey) and DAPI (blue). NEMO-positive FK2 punctae are negative for the K6-affimer (white arrows) suggesting the affimer retains specificity in fluorescence staining of fixed cells.

Figure S6

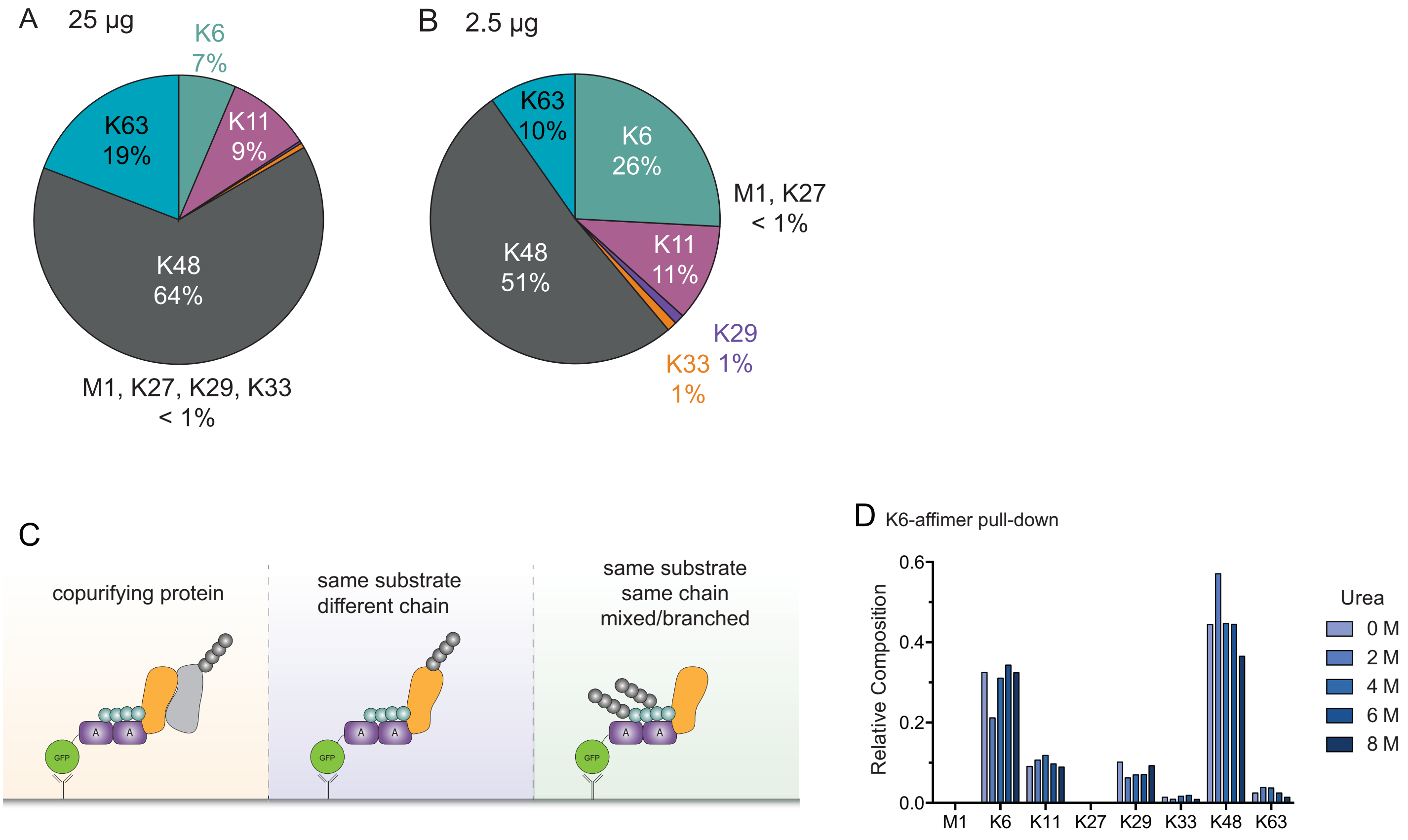

A 25 µg
K6
7%
K11
9%
K63
19%
K48
64%
M1, K27, K29, K33
< 1%
B 2.5 µg
K63
10%
K6
26%
M1, K27
< 1%
K11
11%
K48
51%
K29
1%
K33
1%
C
copurifying protein
same substrate
different chain
same substrate
same chain
mixed/branched
A
GFP
D K6-affimer pull-down
Relative Composition
0.6
0.4
0.2
0.0
M1
K6
K11
K27
K29
K33
K48
K63
Urea
0 M
2 M
4 M
6 M
8 M

Figure S6 (related to Figure 6): ***Enriching Ub linkages using affimers***

A-B) Ub linkage compositions from K6-affimer pull-downs performed with A) 25 µg and B) 2.5 µg of the K6-affimer. **C)** Schematic explaining the different scenarios likely explaining the origin of non K6-chains in K6-affimer pull-downs **D)** Relative Ub chain composition of K6-affimer pull-downs performed in the presence of the indicated concentrations of urea.

Figure S7

A

HUWE1 (aa)	PSMs		
	Rep 1	Rep 2	Rep 3
IPIPLMDYILNVMK (762-775)	-	1	-
FVESILSNNTTDDHCQEFVNQK (776-797)	2	-	-
MVNPTTVLESPHSLPAK (1209-1225)	-	5	3
WITPVLLLIDFYEK (1582-1595)	5	-	
IGEILIQGLTEDMVTVLIR (1742-1760)	2	-	-
SAATSGAGSTTSGVVSGSLGSR (1844-1865)	21	6	6
IVNQPSSLFGSK (2256-2267)	-	3	3
LLGPSAAADILQLSSSLPLQSR (2580-2601)	2	-	-
LLVGNDDVHIIAR (2606-2618)	3	-	-
AGSSTPGDAPPAVAEVQGR (2895-2903)	3	11	11
LLSLISIALPENK (3456-3468)	15	9	5

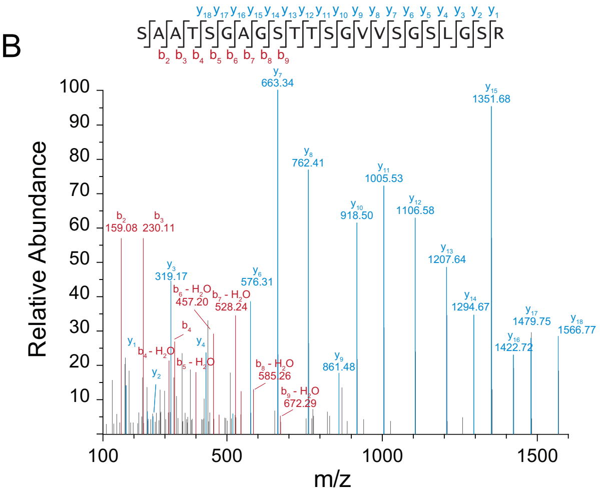

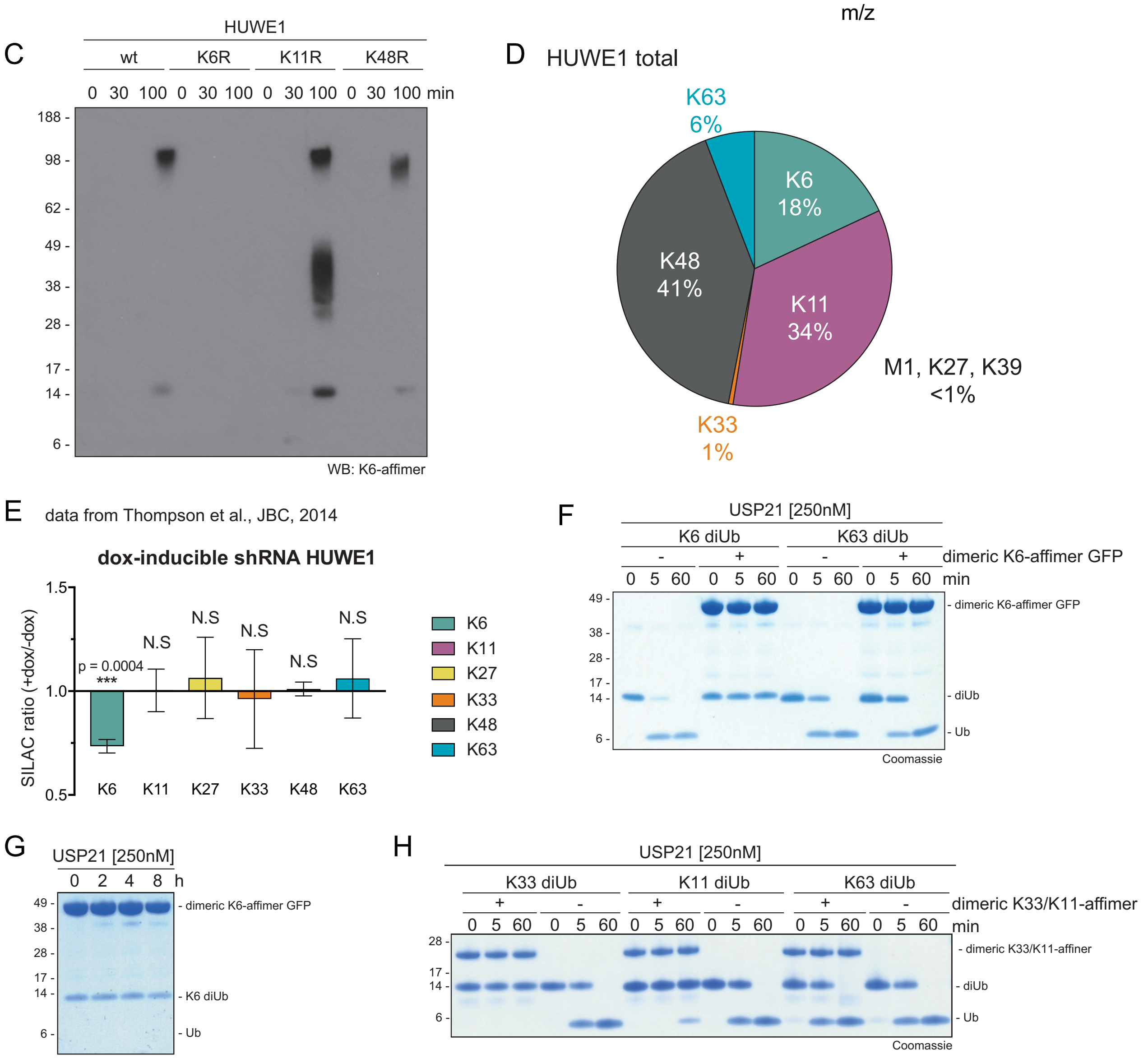

Figure S7 (related to Figure 7): HUWE1 assembles K6 chains *in vitro* and *in vivo*

A) Table summarizing the HUWE1 peptides identified in three replicate K6-affimer pull-down experiments with the corresponding number of PSMs. **B)** MS/MS fragmentation spectrum for one HUWE1 peptide (aa 1844-1865). **C)** Western blot with the K6-affimer of an assembly reaction with recombinant HUWE1 and different Ub mutants. **D)** Ub linkage composition of HUWE1-assembled Ub chains. **E)** SILAC ratios of induced vs. uninduced HUWE1 knockdown cell lines. Only singly modified peptides were analyzed. Data extracted and analyzed from Thompson et al., 2014. Error bars represent mean ± standard deviation. *** $p < 0.001$; N.S not significant as determined by a one sample, two-tailed *t* test. **F)** USP21 deubiquitinase assay in the presence or absence of GFP-tagged K6-affimer for K6 and K63 diUb. **G)** As in D, but with longer time points and only for K6 diUb. **H)** As in F but with K33/K11-affimer and for K33, K11 and K63 diUb.
