# Supplementary material for: Ubiquitin Linkage-Specific Affimers Reveal Insights into K6-Linked Ubiquitin Signaling
Source: Mol Cell. 2017 Oct 5;68(1):233–246.e5. doi: 10.1016/j.molcel.2017.08.020 (PMC5640506; doi:10.1016/j.molcel.2017.08.020)
Supplement: Document S2. Article plus Supplemental Information [file mmc2.pdf]

Resource

Molecular Cell

Ubiquitin Linkage-Specific Affimers Reveal Insights into K6-Linked Ubiquitin Signaling

Graphical Abstract

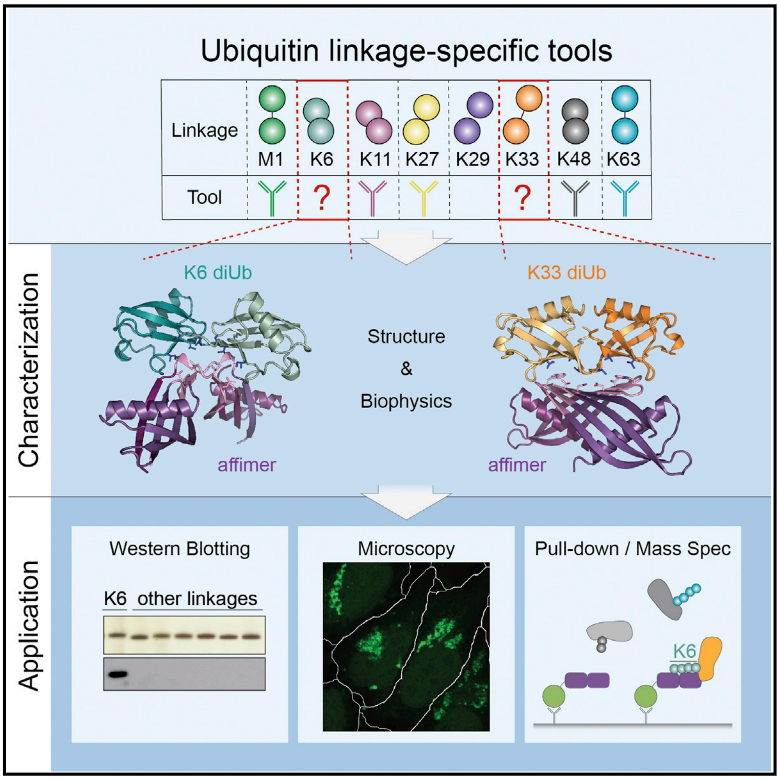

Authors

Martin A. Michel, Kirby N. Swatek, Manuela K. Hospenthal, David Komander

Correspondence

dk@mrc-lmb.cam.ac.uk

In Brief

Michel et al. describe linkage-specific tools for the study of atypical K6 and K33/K11 ubiquitin linkages. Affimers have applications in western blotting, confocal microscopy, and pull-downs. Using a K6-specific affimer, they reveal that HUWE1 is a major source of cellular K6 chains and decorates Mfn2 with K6 chains.

Highlights

- Respective linkage-specific affimers recognize K6- or K33-/K11-linked chains
- Structures of affimer:diUb complexes reveal mechanisms of linkage specificity
- Improved affimers can be used in western blotting, confocal microscopy, and pull-downs
- Pull-downs with K6-specific affimers reveal HUWE1 to be a major K6 ligase in cells

Michel et al., 2017, Molecular Cell *68*, 233–246
October 5, 2017 © 2017 The Authors. Published by Elsevier Inc.
http://dx.doi.org/10.1016/j.molcel.2017.08.020

CellPress

Ubiquitin Linkage-Specific Affimers Reveal Insights into K6-Linked Ubiquitin Signaling

Martin A. Michel,[1] Kirby N. Swatek,[1] Manuela K. Hospenthal,[1,2] and David Komander[1,3,*]
[1]Division of Protein and Nucleic Acid Chemistry, MRC Laboratory of Molecular Biology, Francis Crick Avenue, Cambridge CB2 0QH, UK
[2]Present address: Institute of Structural and Molecular Biology, University College London and Birkbeck, Malet Street, London WC1E 7HX, UK
[3]Lead Contact
*Correspondence: dk@mrc-lmb.cam.ac.uk
http://dx.doi.org/10.1016/j.molcel.2017.08.020

SUMMARY

Several ubiquitin chain types have remained unstudied, mainly because tools and techniques to detect these posttranslational modifications are scarce. Linkage-specific antibodies have shaped our understanding of the roles and dynamics of polyubiquitin signals but are available for only five out of eight linkage types. We here characterize K6- and K33-linkage-specific "affimer" reagents as high-affinity ubiquitin interactors. Crystal structures of affimers bound to their cognate chain types reveal mechanisms of specificity and a K11 cross-reactivity in the K33 affimer. Structure-guided improvements yield superior affinity reagents suitable for western blotting, confocal fluorescence microscopy and pull-down applications. This allowed us to identify RNF144A and RNF144B as E3 ligases that assemble K6-, K11-, and K48-linked polyubiquitin *in vitro*. A protocol to enrich K6-ubiquitinated proteins from cells identifies HUWE1 as a main E3 ligase for this chain type, and we show that mitofusin-2 is modified with K6-linked polyubiquitin in a HUWE1-dependent manner.

INTRODUCTION

Polyubiquitination of proteins is an important posttranslational modification that can lead to a variety of cellular outcomes. The best-studied role of ubiquitination is as a proteasomal degradation tag, but ubiquitin (Ub) also has many non-degradative roles (Swatek and Komander, 2016; Yau and Rape, 2016). This versatility originates in part from the ability of Ub to form distinct polyUb chains. Ub can be ubiquitinated at any of eight primary amines (on the N-terminal amino group of M1 or the side chains of K6, K11, K27, K29, K33, K48, and K63), and all eight Ub linkage types co-exist in cells (Peng et al., 2003). As a consequence, Ub chains have a vast array of architectures.

Cells utilize distinct Ub linkages in different signaling contexts, which invokes the need for linkage specificity at three levels: assembly; recognition; and disassembly. Linkage-specific assembly of polyUb is mediated by a subset of E2-conjugating enzymes and E3 Ub ligases (Ye and Rape, 2009; Zheng and Shabek, 2017). Particularly interesting are E3 enzymes of the HECT and RBR families, because these often assemble Ub chains in a linkage-specific fashion. Once a Ub chain is formed, it is recognized by Ub-binding domains (UBDs) (Husnjak and Dikic, 2012), and this can occur in a linkage-specific manner. Selective UBDs for five out of the eight linkage types are known to date (Swatek and Komander, 2016; Yau and Rape, 2016). Finally, deubiquitinases (DUBs) disassemble the Ub chains, and some cleave chains with high linkage selectivity (Clague et al., 2013; Mevissen and Komander, 2017).

The most abundant Ub chain types are K48-linked chains, which target proteins for proteasomal degradation, and K63-linked chains, which have non-degradative roles in intracellular trafficking, kinase signaling, DNA damage response, and other processes. The remaining six linkages, so-called "atypical" chains, are less abundant, but first roles are emerging (Swatek and Komander, 2016; Yau and Rape, 2016). For example, K6- and K33-linked chains were shown to increase after DNA damage (Elia et al., 2015), and earlier data linked K6 linkages to the E3 Ub ligase BRCA1 (Morris and Solomon, 2004; Wu-Baer et al., 2003). K6 chains assembled by the RBR E3 Ub ligase Parkin were also shown to be important for mitophagy (Durcan et al., 2014; Ordureau et al., 2014, 2015), which is antagonized by the K6-selective, mitochondrial DUB USP30 (Bingol and Sheng, 2016).

A main reason that less abundant chain types are still understudied is the current lack of tools to enable linkage-specific detection. Linkage-specific antibodies have been generated for five of the eight Ub chain types (Matsumoto et al., 2010, 2012; Newton et al., 2008; Figure 1A) and were instrumental to study K48-/K63-chain editing (Newton et al., 2008), the importance of K11 chains in cell cycle regulation (Matsumoto et al., 2010), and M1 chains in inflammation (Elliott, 2016), respectively. Especially for linkages without known endogenous regulators, the generation of linkage-specific detection reagents is crucial.

Here, we describe linkage-specific Ub affinity reagents for K6- and K33-/K11-linked chains, which were derived from non-antibody protein "affimer" scaffolds. Crystal structures of affimers bound to their cognate diUb reveal how they mimic naturally occurring linkage-specific UBDs and explain their linkage specificity. Guided by these structures, initial affimers were improved,

© 2017 The Authors. Published by Elsevier Inc.
This is an open access article under the CC BY license (http://creativecommons.org/licenses/by/4.0/).

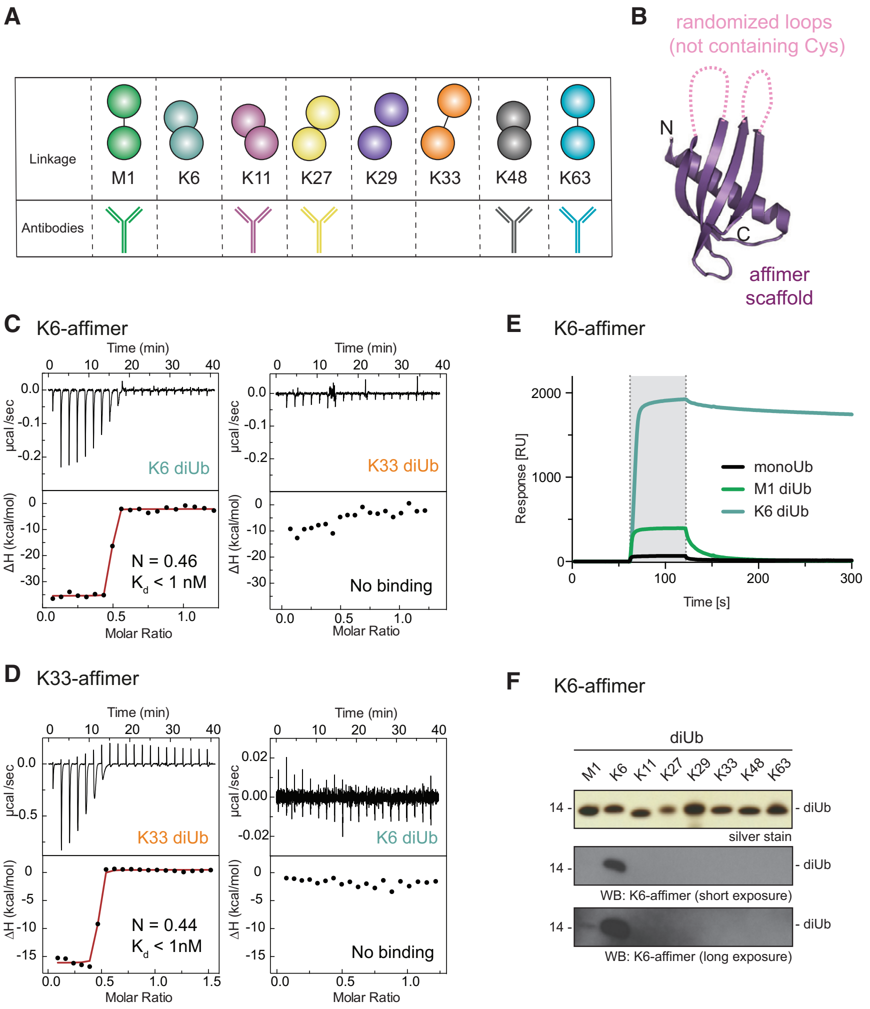

Figure 1. Linkage Specificity of Affimers

(A) Overview of Ub linkages with available linkage-specific antibodies.

(B) Structure of general affimer scaffold (purple) with randomized loops (pink) that is used in phage display (PDB: 4N6U).

(C) ITC titration of K6 affimer (5 µM; in cell) against K6 diUb (30 µM in syringe; left) and K33 diUb (30 µM in syringe; right), showing plots of raw heat (top) and derived isotherms (bottom) with fitted curve (red).

(D) As in (C) but with K33 affimer.

(E) SPR measurements of K6 affimer (10 µM) injected onto a chip where monoUb (black), M1 diUb (green), and K6 diUb (cyan) are immobilized.

(F) Silver stain input of purified diUbs and western blot with biotinylated K6 affimer.

See also Figure S1.

and the resulting reagents are shown to be useful in a variety of applications. Using affimers in western blotting in combination with mass spectrometry reveals that the RBR E3 ligases RNF144A and RNF144B assemble predominantly K6-, K11-, and K48-linked chains *in vitro*. We further show that these affimers specifically recognize their cognate linkage also in a cellular background. In pull-downs using affimers to enrich K6 chains from cells, we identify the HECT E3 ligase HUWE1 and go on to show that HUWE1 also assembles K6-, K11-, and K48-linked chains *in vitro*. Interestingly, HUWE1$^{-/-}$ or HUWE1 knockdown cells show significantly reduced levels of K6 chains, indicating that HUWE1 is a major source of cellular K6 chains. Further, we show that mitofusin-2 (Mfn2), a known substrate of HUWE1, is modified with K6 chains in a HUWE1-dependent manner.

RESULTS

Linkage-Specific Tools for Atypical Ubiquitin Chains

Ub-specific antibodies are notoriously difficult to generate in animals due to the high identity of Ub between species, and as a consequence, most available linkage-specific antibodies (Figure 1A) were selected using phage display (Matsumoto et al., 2010, 2012; Newton et al., 2008). Affimer technology provides an alternative route to generate specific high-affinity reagents. Affimers are 12-kDa non-antibody scaffolds based on the cystatin fold (Figure 1B; Tiede et al., 2014, 2017), in which randomization of surface loops enables the generation of large (10^{10}) libraries, against which epitopes can be screened and binders can be selected. We characterized affimers against K6- and K33-linked diUb generated by Avacta (Wetherby, UK), and performed isothermal titration calorimetry (ITC) measurements on both affimers against K6- and K33-linked diUb. We found that the K6 affimer bound tightly to K6 diUb, whereas no binding could be detected to K33 diUb (Figures 1C and S1A–S1C). Similarly, the K33 affimer bound K33 diUb but failed to detectably interact with K6 diUb as judged by ITC (Figures 1D and S1C). Interestingly, the ITC measurements indicated the formation of a 2:1 affimer:diUb complex (n = 0.46 for K6 affimer and n = 0.44 for the K33 affimer), suggesting that the affimer dimerized for diUb binding. Qualitative kinetic analysis by surface plasmon resonance (SPR) on the K6 affimer showed that linkage specificity is achieved through very low off rates only for the cognate diUb (Figure 1E). We next tested site-specifically biotinylated affimers in western blotting against diUb of all linkage types. Indeed, the K6 affimer detected K6 diUb with high linkage specificity (Figure 1F) and only showed weak off-target recognition of other chain types, although cross-specificity was more pronounced with tetraUb (Figure S1D). In contrast, the K33 affimer did not lead to any detectable signal by western blotting (Figure S1E). The discrepancy in K33 linkage detection in ITC and western blotting is likely due to the different concentrations used in these experiments (50 nM in western blotting

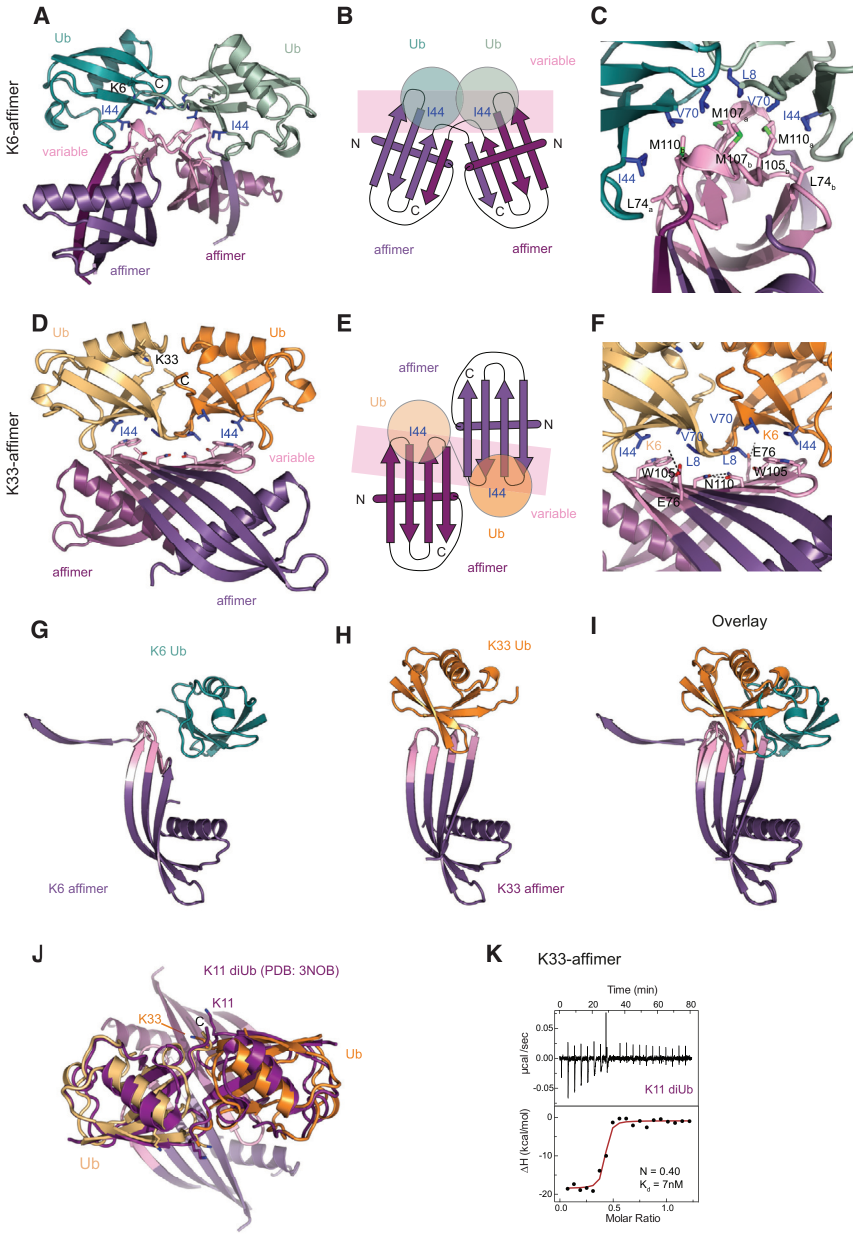

Figure 2. Structures of Linkage-Specific Affimers with Their Cognate diUbs

(A) Crystal structure of K6 affimer (shades of purple) bound to K6 diUb (shades of cyan) at 2.5 Å resolution. The variable loops (pink) contact the Ile44 patch (blue) on Ub.

(B) Schematic of structure in (A) with indicated N and C termini.

(C) Close up of the interaction in the K6 affimer:K6 diUb structure, showing hydrophobic interactions mediated by the variable loops (pink) of the affimer.

(D) Crystal structure of the K33 affimer (shades of purple) bound to K33 diUb (shades of orange) at 2.8 Å resolution. The affimer dimerizes distinctly from the K6 affimer (see A) to bind diUb. Variable regions (pink) interact with the Ile44 patch (blue) of the Ub moieties.

(E) As in (B) but for the K33 affimer:K33 diUb structure.

(F) As in (C) but for the K33 affimer:K33 diUb structure.

(G–I) Structures of one affimer bound to Ub, showing relative orientations of the bound Ub, as observed in the K6 affimer (G) and K33 affimer (H) structures and in the overlay (I).

(J) Overlay of the K33 affimer:K33 diUb structure with previously determined K11 diUb structure (magenta; PDB: 3NOB).

(K) ITC measurement of K33 affimer (5 μM in cell) against K11 diUb (30 μM in syringe).

See also Figure S2 and Table 1.

and 5 μM in ITC), which could affect, for example, the dimerization equilibrium.

Crystal Structures Explain Affimer Specificity

To understand the observed linkage specificities, we determined crystal structures of the K6 and K33 affimers bound to their cognate diUb at 2.5 Å and 2.8 Å resolution, respectively (Figures 2 and S2; Table 1). Both complexes show a conceptually similar interaction between affimers and diUb: each affimer molecule binds one Ub molecule and the affimer dimerizes to bind the two Ub moieties of a diUb in a linkage-specific manner (Figures 2A–2F and S2A–S2C). The variable loops are responsible for dimerization as well as Ub recognition, and specific dimerization provides two binding sites for Ub I44 patches with a defined distance and relative orientation, leading to specific, high-affinity recognition. Other diUb linkages can only be bound by one affimer at a time, which significantly reduces the affinity. This is reminiscent of naturally occurring UBDs that provide two binding surfaces, and only the cognate linkage is able to occupy both simultaneously (Komander and Rape, 2012).

Individually, however, the structures of affimer-bound diUb complexes are surprisingly distinct (Figures 2A–2I). In the K6 affimer, the first variable loop extends the existing β strands, whereas the second variable loop extends into an α-helical turn (Figure S2D) that bridges to the second affimer molecule and engages in a strand swap of the last β strand. This leads to a symmetric dimer, similar to what has been found for naturally occurring cystatins (Janowski et al., 2001; Figure S2E). L74, I105, M107, and M110 in the variable loops create a hydrophobic surface that interacts with the I44 patch of Ub (L8, I44, and V70; Figure 2C), and four salt bridges further strengthen this interaction (Figure S2C).

In the K33 affimer, the mode of dimerization is different (Figures 2D–2F). Both variable loops extend the existing β strands, which leads to the formation of an intermolecular β sheet

Table 1. Data Collection Statistics

	K6 Affimer: K6 diUb	K33 Affimer: K33 diUb	K33 Affimer: K33 diUb
	PDB: 5OHM	PDB: 5OHV	PDB: 5OHL
Data Collection			
Beamline	ESRF ID23-2	Diamond I04	Diamond I04-1
Space group	*P* 1	*H* 3	$P\,2_1$
a, *b*, *c* (Å)	60.5, 69.7, 99.3	120.3, 120.3, 69.9	55.9, 149.6, 73.8
α, β, γ (°)	79.8, 79.8, 83.1	90, 90, 120	90, 110.4, 90
Wavelength (Å)	0.872900	0.979490	0.917410
Resolution (Å)	48.23–2.50 (2.59–2.50)	60.14–2.80 (2.90–2.80)	48.46–3.80 (3.936–3.80)
R_{merge}	0.06 (0.26)	0.235 (0.794)	0.237 (0.779)
$I/\sigma I$	8.2 (2.4)	5.0 (2.0)	3.8 (1.5)
$CC_{1/2}$	0.996 (0.791)	0.978 (0.679)	0.962 (0.665)
Completeness (%)	98.4 (97.5)	100 (100)	97.9 (98.5)
Multiplicity	1.8 (1.8)	7.0 (7.1)	3.3 (3.4)
Refinement			
Resolution (Å)	48.23–2.50	60.14–2.80	48.46–3.80
No. reflections	53,198	9,279	10,962
R_{work}/R_{free}	20.0/22.7	18.5/22.6	23.45/28.51
Clashscore	5.63	7.70	11.63
No. Atoms			
Protein	10,490	2,587	7,123
Ligand/ion	57	34	36
Water	105	15	0
B Factors			
Wilson *B*	47.74	56.61	83.78
Protein	56.63	61.65	126.41
Ligand/ion	55.09	84.34	78.37
Water	46.38	50.41	–
Root-Mean-Square Deviations			
Bond lengths (Å)	0.005	0.004	0.003
Bond angles (°)	0.996	0.85	0.67
Ramachandran statistics (favored/allowed/ outliers)	98.7/1.3/0	99.1/0.9/0	97.9/2.1/0

Numbers in brackets are for the highest resolution bin.

(Figure S2D). Ub interactions are mediated by the elongated β sheet and are centered on W105 that contacts the I44 patch (Figure 2F). Additionally, E76 forms a salt bridge with K6 of Ub (Figure 2F). The structure also indicates how the affimer could bind to longer K33 polymers (Figure S2F). Finally, we solved a 3.8-Å structure of the K33 affimer bound to K33 diUb in a different space group, which shows the same overall orientation and interactions (Figure S2G).

Differently linked Ub chains adopt distinct conformations, and binding partners select a suitable one from the population of conformations each chain can adopt (Ye et al., 2012). Crystal structures have captured some of these conformational states and reveal what a chain type can look like. Interestingly, whereas the affimer-bound K6 diUb does not resemble previous K6 crystal structures (Hospenthal et al., 2013; Virdee et al., 2010; Figure S2H), the affimer-bound K33 diUb superimposes well with reported K33 diUb crystal structures (Figure S2I; Kristariyanto et al., 2015; Michel et al., 2015). The latter also closely resembles a conformation adopted by K11 diUb (Bremm et al., 2010; Matsumoto et al., 2010; Figure 2J), suggesting that the K33 affimer could be cross-specific with K11 polyUb. ITC measurements indeed confirm that the K33 affimer also dimerizes to bind K11 diUb tightly, with affinities slightly weaker than the K33 diUb but still in the low nM range (Figure 2K). As phage display selections and ITC measurements were all done in solution, this suggests that the observed conformations for K6, K33, and also K11 diUb exist in solution and that the affimer selects this particular conformation from the conformational ensemble.

Dimerized Affimers Show Improved Binding Characteristics

The dimeric states of affimers in solution and in the crystal structures were further confirmed by size-exclusion chromatography with multi-angle light scattering (SEC-MALS). This showed that

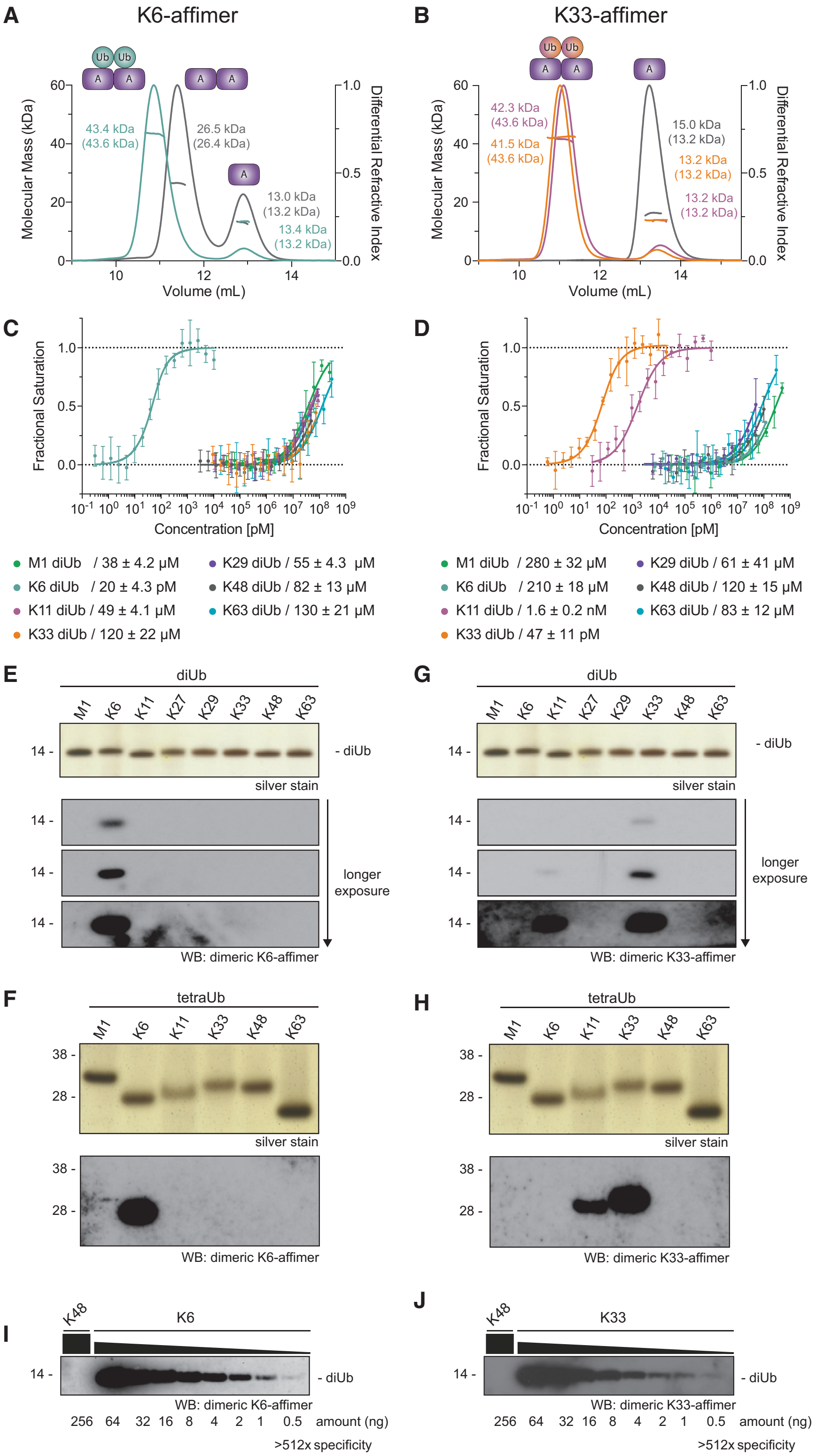

Figure 3. Affimers Dimerize to Achieve Linkage Specificity

(A) SEC-MALS analysis of K6 affimer alone (gray) and with a 0.95 molar equivalent of K6 diUb (cyan). Observed molecular masses are shown with expected molecular masses in brackets.

(B) As in (A) but for K33/K11 affimer alone (gray) and with K33 diUb (orange) or K11 diUb (magenta).

(C) Microscale thermophoresis (MST) binding assay of dimerized K6 affimer against differently linked diUb. Data were fitted to a single-site binding model accounting for ligand depletion. Error bars represent mean ± SD.

(D) As in (C) but for the K33/K11 affimer.

(E) Silver stain input and western blot of purified diUbs with biotinylated, dimerized K6 affimer.

(F) As in (E) but for tetraUb.

(G) As in (E) but with the dimerized K33/K11 affimer. The silver stain input gel is the same as in (E).

(H) As in (F) but for tetraUb. The silver stain input gel is the same as in (F).

(I) Indicated amounts of K48 and K6 diUb were probed with dimerized K6 affimer.

(J) As in (I) but with K48 and K33 diUb and probed with the dimerized K33/K11 affimer.

See also Figure S3.

the K6 affimer alone is in a concentration-dependent monomer-dimer equilibrium, which is shifted fully toward the dimer by addition of diUb (Figure 3A). The strand swap observed in the K6 affimer crystal structure is likely responsible for the relatively stable dimer in the absence of diUb. In contrast, the K33/K11 affimer is monomeric and only dimerizes upon diUb binding (Figure 3B). This suggests that this affimer requires high concentrations to work, likely explaining the lack of signal in western blotting (Figure S1E).

Biophysical and structural data collectively indicated that a covalent, constitutive affimer dimer may have improved binding characteristics. To test this, we truncated and fused the affimers, as guided by the structure, to generate tandem repeat dimeric affimers (Figure S3A), and we characterized these dimerized versions biophysically. Binding equilibria were achieved within 2 hr and 1 hr for the K6 and K33/K11 affimer, respectively, even at concentrations of 5 nM that are comparable to or below concentrations used in subsequent applications (Figures S3B and S3C; see below). The binding affinities of these affimers for differently linked diUb were determined using fluorescence polarization (FP) and microscale thermophoresis (MST) assays. We found that the K6 affimer bound the cognate K6 diUb with very high affinity (K_d ~20 pM) and has negligible affinity for the other diUb linkages (K_d > 40 μM; Figures 3C and S3D). Similarly, the K33/K11 affimer binds K33 and K11 diUb tightly (with a K_d of 47 pM and 1.6 nM, respectively) and again showed

little binding to non-cognate diUbs (K_d > 50 μM; Figures 3D and S3E). In western blotting, the dimerized K6 affimer still recognized K6 polyUb specifically with very little background even at long exposures (Figures 3E and 3F). Furthermore, the dimerized K33/K11 affimer started to work in western blotting and detected K33, and to an ~4-fold lesser extent K11 diUb, consistent with affinity data (Figures 3G, 3H, and S3F). Western blotting with diUb titration suggests that the K6 and K33/K11 affimers prefer their cognate diUb ≥1,000-fold over other diUb linkages (Figures 3I, 3J, S3G, and S3H). Due to the superior binding properties of the dimerized affimers, all subsequent experiments were performed with these improved versions.

Affimers Faithfully Detect Longer Ub Chains and Reveal E3 Ligase Specificities

To further characterize and exploit affimers, we used them to identify chain types assembled by E2 and E3 enzymes. To test this, Ub chains were assembled with the K11-specific E2 UBE2S (Bremm et al., 2010), the K11-/K33-specific HECT E3 ligase AREL1 (Michel et al., 2015), and the K6-/K48-specific HECT-like E3 NleL (Hospenthal et al., 2013). HECT, HECT-like, and RBR E3s dictate the type of Ub linkage they assemble independently of the E2 used (Zheng and Shabek, 2017), and for these families of E3 ligases, the E2 only serves to charge Ub onto the active site Cys. The E2 enzyme UBE2L3 is specific for this trans-thioesterification reaction (Wenzel et al., 2011) and works well with HECT and RBR E3s.

UBE2S assembles K11 chains, and these were recognized by the K33/K11 affimer (Figure 4A). Whereas some conjugates were still formed using Ub K11R, the K33/K11 affimer did not recognize these products (Figure 4A), suggesting that these are not K33 conjugates. Similarly, the K33/K11 affimer also detected products of AREL1, which assembles mostly K11 and K33 chains with wild-type Ub, independently of which E2 is used (Michel et al., 2015; Figure 4B). The signal slightly increased using a K11R Ub mutant and was reduced with a K33R Ub mutant (Figure 4B), in agreement with the preferred detection of K33 chains over K11 chains (Figure S3F). NleL is a HECT-like effector E3 ligase from *E. coli* O157:H7 that assembles mixed and branched K6- and K48-linked chains *in vitro* (Hospenthal et al., 2013), and these chains were recognized by the K6-specific affimer (Figure 4C). Chains assembled with a Ub K6R mutant to prevent the formation of K6 chains yielded no K6 signal, whereas using Ub K48R increased the signal (Figure 4C), consistent with linkage-specific detection of K6 chains.

Next, we set out to characterize the products of ligases with unknown linkage specificities. Many RBR-type E3 ligases, including HOIP and Parkin, assemble atypical Ub chain types, but several others have remained unstudied. We tested the RBR E3 ligases RNF144A and RNF144B, both of which are uncharacterized with regards to their linkage specificity. The proteins are highly homologous, and both comprise an RBR domain and a C-terminal transmembrane domain. The isolated RBR domain of both RNF144A and RNF144B can be expressed in soluble form in *E. coli* and together with the E2 UBE2L3, they assemble free as well as conjugated Ub chains *in vitro* (Figure 4D). Western blotting revealed a strong signal with the K6- as well as with the K33-/K11-specific affimers, indicating that both ligases assembled these atypical chain types (Figure 4D). This warranted more detailed analysis by AQUA-based mass spectrometry (MS), which showed that RNF144A and RNF144B assembled predominantly K11 (47% and 62%, respectively) but also K6 (22% and 10%), K48 (25% and 10%), and K63 chains (Figures 4E, 4F, S4A, and S4B). This was further confirmed by linkage-specific antibodies (Figures S4C and S4D).

Affimers Detect K6 Chains in Cells

Having shown that affimers are linkage specific *in vitro*, we set out to test them in a cellular context. We focused our studies on the K6 affimer as it was more specific, and K6 chains had been implicated in intriguing biological processes. The abundance of K6-linked chains is reportedly low at <1% of all linkages (Dammer et al., 2011), which provided a challenging opportunity to study this chain type.

First, we generated a stable T-REx 293 cell line, in which a full-length, hemagglutinin (HA)-tagged version of the bacterial effector NleL is expressed in an inducible manner. Consistent with the low abundance of K6 chains, western blotting of whole-cell lysate (WCL) with the K6 affimer yielded no signal. This changed upon induced expression of NleL, which did not cause a global increase in total Ub conjugates but resulted in a strong signal with the K6 affimer (Figure 5A). Enriching all Ub conjugates using TUBEs (Hjerpe et al., 2009) improved the signal further and, interestingly, also enabled detection of K6 chains in non-induced cells (Figure 5A). This shows that the K6 affimer can be used to detect K6 chains in a cellular context and, with polyUb enrichment, even at endogenous levels.

Similar results were obtained with HEK293 cells, where K6 linkages could not be detected in WCL, even after 1 hr treatment with the proteasome inhibitor MG132 (Figure 5B). TUBE-mediated enrichment of polyUb enabled robust detection of endogenous K6 chains (Figure 5B). Consistent with previous reports, UV radiation (40 J/m^2 with 1 hr recovery in medium containing 10 μM MG132, according to Elia et al., 2015) or mitochondrial decoupling (10 μM CCCP for 1 hr in presence of 10 μM MG132) increased the K6 signal in TUBE pull-downs 2- to 3-fold (Figure 5B). This is consistent with recent data suggesting a 3- to 4-fold increase in K6 linkages upon DNA damage (Elia et al., 2015) and 2- to 8-fold increase upon mitochondrial depolarization (Cunningham et al., 2015; Ordureau et al., 2014). Importantly, whereas K6 linkages account for only a small fraction of linkages present, they are faithfully detected by the K6 affimer.

PINK1/Parkin-mediated mitophagy is emerging as a robust system for Ub studies. Parkin as well as the mitochondrial DUB USP30 regulate mitophagy in part by regulating K6-linked chains on mitochondria (Cunningham et al., 2015; Durcan et al., 2014; Ordureau et al., 2014; Gersch et al., 2017). For this, HeLa Flp-In T-REx cells inducibly expressing wild-type (WT) or catalytically inactive (C431S) Parkin were depolarized with oligomycin/antimycin A (O/A), leading to robust Parkin-dependent ubiquitination of mitochondrial proteins, including TOM20 (Figure 5C; Ordureau et al., 2014; Sarraf et al., 2013). Importantly, whereas there is no overall change in cellular Ub

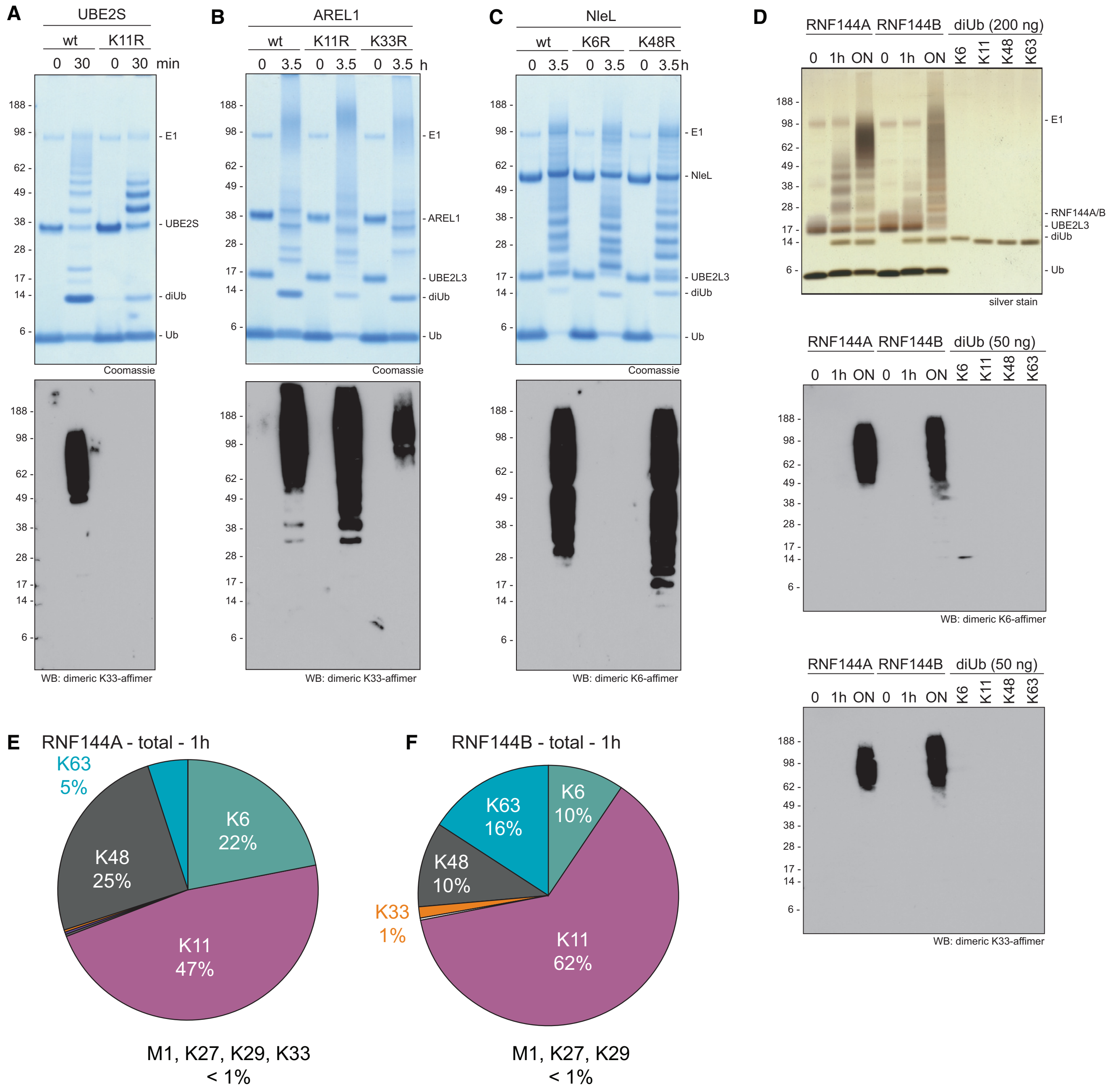

Figure 4. *In Vitro* Applications of Affimers

(A) *In vitro* assembly reaction of the E2 UBE2S with Ub WT and Ub K11R with Coomassie (top) or blotted with the K33/K11 affimer (bottom).

(B) *In vitro* assembly reaction of the HECT E3 AREL1 with Ub WT, Ub K11R, and Ub K33R stained with Coomassie (top) and probed by western blotting with the K33/K11 affimer (bottom). Longer chains are preferentially detected, probably due to avidity effects.

(C) *In vitro* assembly reaction of the HECT-like E3 NleL with Ub WT, Ub K6R, and Ub K48R stained with Coomassie (top) or probed by western blotting with the K6 affimer (bottom).

(D) *In vitro* Ub chain assembly reactions for RNF144A and RNF144B, with Ub WT, alongside recombinant diUb standards on silver stain (top) and probed with the K6 affimer (middle) and the K33/K11 affimer (bottom).

(E) AQUA-MS-derived linkage composition of RNF144A-assembled total Ub chains at a 1 hr time point.

(F) As in (E) but for RNF144B.

See also Figure S4.

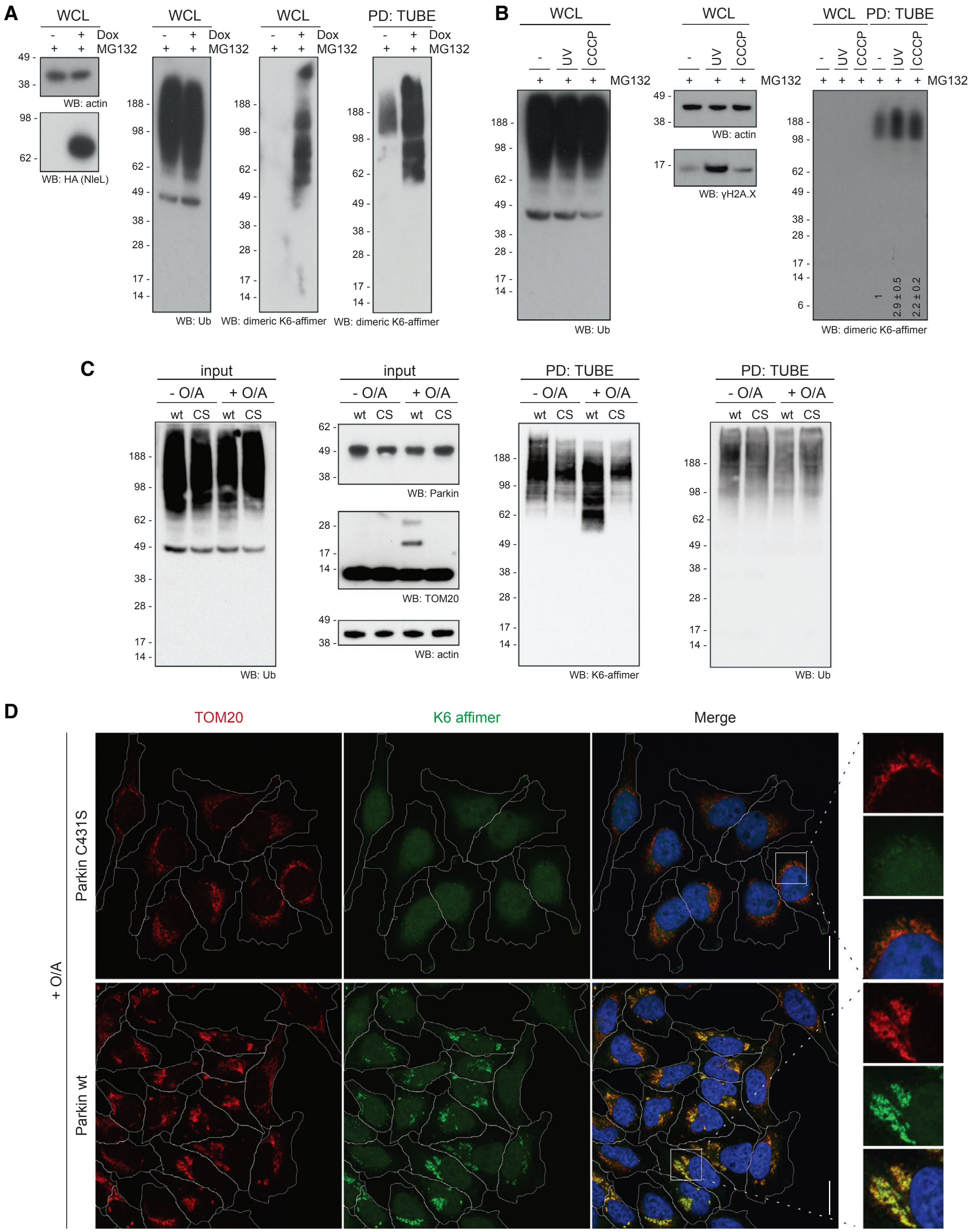

Figure 5. *In Vivo* Applications of Affimers

(A) HA-NleL 293 T-Rex cells were induced with 1 μg/mL doxycycline for 12 hr or left untreated. Whole-cell lysate (WCL) blots are shown for actin, HA(-NleL), Ub, and K6 chains. Western blots with the K6 affimer are also shown after Ub enrichment using TUBEs.

(B) TUBE-PD of HEK293 cells after 1 hr of MG132 (10 μM) without further treatment (–) or with additional UV (40 J/m^2) or CCCP treatment (10 μM for 1 hr) and subsequently blotted with the K6 affimer. Input controls are shown for total Ub and actin and γH2AX. The relative signal increase from two experiments is shown below the respective lanes.

(legend continued on next page)

(Figure 5C), TUBE pull-down and blotting with the K6 affimer reveals an increase in K6 chains in a depolarization- and Parkin-dependent manner (Figure 5C). Blotting of the TUBE pull-down for total Ub shows equal amounts of Ub in all lanes (Figure 5C), further demonstrating that the differential signal observed in the K6 affimer blot is indeed due to K6 chains.

To further demonstrate the accumulation of K6 chains on mitochondria during mitophagy, we tested the utility of the K6 affimer in confocal fluorescence microscopy. Using site-specifically Alexa-488-labeled, dimeric K6 affimer on fixed, permeabilized cells, we observed that the K6 affimer displayed diffuse, mostly nuclear localization in untreated cells (Figure S5A). However, upon depolarization using O/A, the K6 affimer relocalized to mitochondria in WT Parkin-reconstituted cells, but not in cells expressing Parkin C431S (Figures 5D and S5B). This was accompanied by perinuclear clustering of depolarized mitochondria (Figure 5D), which has been previously reported (Narendra et al., 2010). Similar staining can be achieved with a total Ub conjugates antibody (FK2) because under these conditions, mitochondria are highly ubiquitinated (Ordureau et al., 2014; Figure S5B). However, the FK2 antibody also labels additional sites, e.g., in nuclei, that do not co-stain with K6 affimer (Figure S5B), suggesting that the affimer retains at least some of its specificity under these conditions. To further test this, we treated cells with tumor necrosis factor alpha (TNF-α) to induce signaling complexes with high-density Ub modifications comprising K63 and M1 linkages (Tarantino et al., 2014). These structures are labeled with FK2 anti-Ub and anti-NEMO antibodies, but not with the K6 affimer, indicating that these Ub-rich structures lack detectable amounts of K6 chains. This further suggests that the affimer has negligible off-target binding when used at suitable concentrations.

Using Affimers as Affinity Matrices for Mass Spectrometry

In pull-down applications, the K6 affimer selects its cognate chain type from a mixture of K6, K48, and K63 tetraUb and shows no detectable off-target binding in absence of K6 linkages (Figure 6A). Similarly, the K33/K11 affimer quantitatively binds K33 and K11 diUb but fails to pull down K6 diUb (Figure 6B). This prompted us to establish a MS-compatible protocol for the enrichment of K6 linkages, in order to identify proteins modified with this chain type. PolyUb species captured on a K6 affimer matrix were subjected to AQUA MS analysis to determine the Ub linkage composition. Despite the high specificity observed *in vitro* (Figures 2, 3, 4, and 6A), we were concerned about non-specific polyUb interactions and attempted to adjust the amount of affimer to expected amounts of cellular K6 linkages (Figure 6C). About 0.5% of the proteome of HEK293 cells is Ub, of which about 10% is in chains (Kaiser et al., 2011). K6 linkages account for 0.5% of all Ub linkages in HEK293 cells (Dammer et al., 2011), and assuming that a 10-cm^2 dish of HEK293 cells contains 2 mg total protein, about 500 ng of K6-linked Ub is expected for this amount of cells. We therefore performed affimer pull-downs from a 10-cm^2 dish of HEK293 cell using 250 ng, 2.5 μg, and 25 μg K6 affimer, respectively (Figures 6D, 6E, S6A, and S6B).

At the highest concentration of affimer (25 μg; 100-fold excess over estimated K6 chains), K6 chains are enriched ~10-fold over their total cellular composition (Figures 6D and S6A), enriched from ~0.5% of linkages in cells (as detected in TUBE pull-downs [not shown] or according to Dammer et al., 2011) to 7%. Under these conditions, the majority of Ub linkages were K48 chains (64%), K63 chains (19%), and K11 chains (9%; Figure S6A), reflecting those linkages most abundant in cells.

Rewardingly, reducing the amount of K6 affimers on the resin led to significant enrichment of K6 polyUb over other chain types: at the lowest affimer concentration (250 ng), K6 linkages were enriched 75-fold (enrichment from 0.5% to 37% of linkages detected; Figures 6D and 6E) and were now the second most abundant chain type in the pull-down (after K48 chains at 48%).

The presence of large amounts of other linkage types in the K6 affimer pull-down could have several reasons (Figure S6C). To exclude that other chain types originate from co-purifying proteins, we performed pull-downs in presence of 0–8 M urea. At urea concentrations above 4 M, the amount of Ub detected was strongly reduced (not shown), suggesting that affimer:Ub interactions were not stable at high urea concentrations. Importantly, however, linkage compositions in pull-downs did not change appreciably at any urea concentration (Figure S6D). This strongly suggested that co-purifying chains were attached to the same substrates that also contained K6 linkages, either as separate chains or in form of heterotypic (mixed or branched) chains. To further improve the enrichment of K6 linkages in the pull-down, beads were treated with improved versions of OTUB1 and AMSH (Michel et al., 2015) to remove K48 and K63 chains, respectively. With this setup, K6 chains were the dominant chain type in the pull-down (56%), representing a >100-fold enrichment of K6 linkages in the sample (Figure 6F).

HUWE1 Assembles K6 Chains *In Vitro* and *In Vivo*

In the established K6 enrichment protocols, proteins detected specifically in the affimer pull-down are likely modified with K6-linked chains. We hence performed shotgun proteomics on DUB-treated K6 affimer pull-downs (Figure 6E) to identify K6-polyubiquitinated proteins. Interestingly, the second most enriched protein (after Ub), and the only other protein observed in all three replicates, was the HECT E3 ligase HUWE1 (also known as Mule, ARF-BP1, LASU1, or HECTH9; Figures 7A, S7A, and S7B). HUWE1 is a large (480 kDa) and highly abundant protein, mutations in which cause X-linked mental retardation syndromes (Friez et al., 2016). Moreover, HUWE1 has been intensely studied in context of cancer, where it was assigned pro-oncogenic but also tumor-suppressive functions (Scheffner

(C) Expression of WT or catalytically inactive (C431S) Parkin in HeLa Flp-In cells was induced with 0.2 μg/mL doxycycline for 16 hr. Mitochondria were depolarized with O/A for 2 hr. WCL inputs are shown for total Ub, expressed Parkin, TOM20, and actin. The TUBE-PD was also blotted using the K6 affimer and total Ub.
(D) Confocal fluorescence microscopy images of cells as in (B) stained with K6 affimer (green), TOM20 (red), and DAPI (blue). Cells and a magnified area are outlined in white. Scale bars correspond to 20 μm.
See also Figure S5.

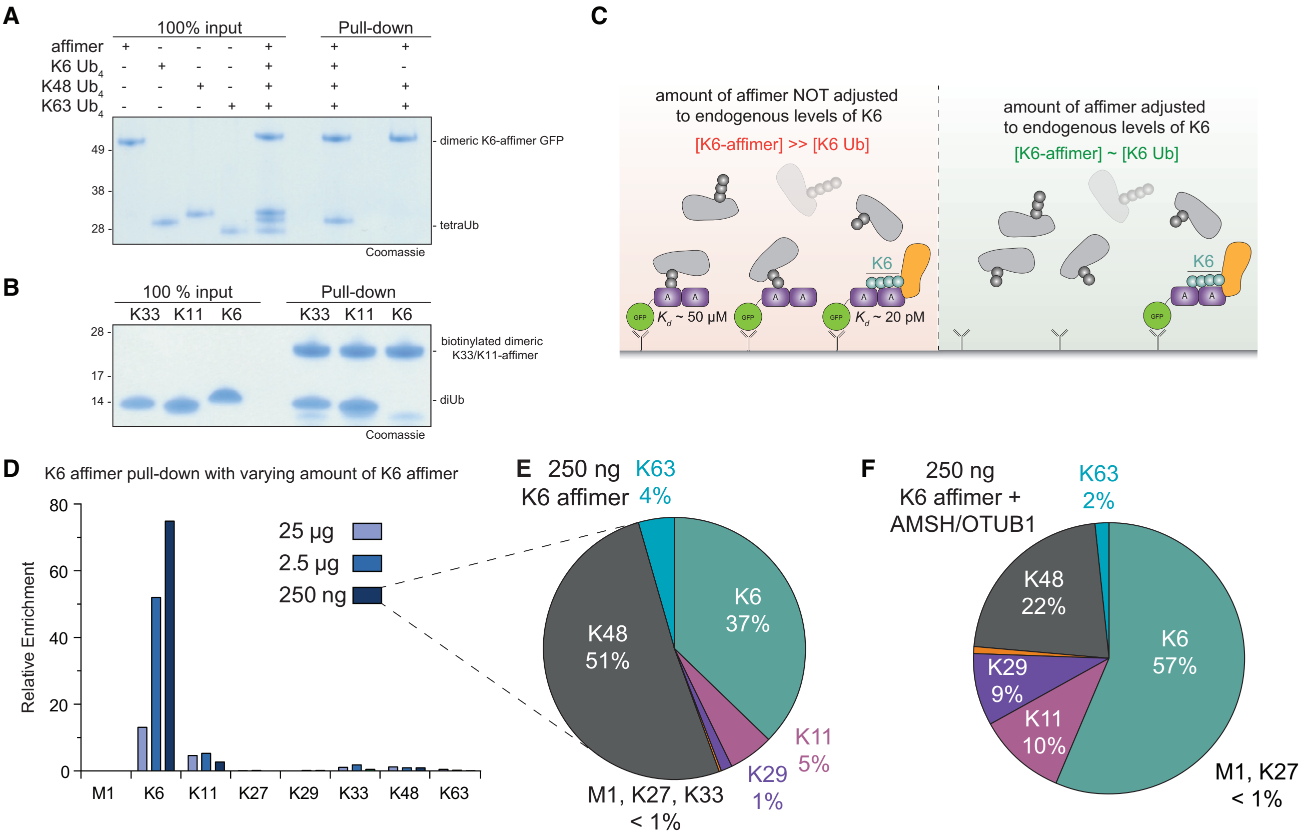

Figure 6. Enriching Ub Linkages Using Affimers

(A) *In vitro*, competition pull-down with dimerized K6 affimer against differently linked tetraUb.

(B) *In vitro* pull-down with biotinylated, dimerized K33/K11 affimer against different diUb linkages.

(C) Schematic of pull-downs with varying amounts of GFP-tagged K6 affimer, either adjusted to expected amounts of K6 chains or in excess thereof.

(D) Relative enrichment of the different chain types after K6 affimer pull-down using different amounts of affimers.

(E) Total chain composition of pull-downs performed in (E) with 250 ng of K6 affimer.

(F) Chain composition of K6 affimer pull-down with 250 ng K6 affimer and treating the pull-downs with 1 μM OTUB1 and 1 μM AMSH for 1 hr on ice.

See also Figure S6.

and Kumar, 2014). Its wide range of substrates implicate the protein in cellular processes, including DNA damage signaling (Choe et al., 2016; Myant et al., 2017; Parsons et al., 2009), cell death signaling (Zhong et al., 2005), and mitochondrial maintenance (Senyilmaz et al., 2015), and most recently in quality control pathways relating to ribosome biogenesis (Sung et al., 2016; Xu et al., 2016). HUWE1 is heavily posttranslationally modified, featuring numerous phosphorylation and ubiquitination sites. The finding that it is pulled down by the K6 affimers suggests that at least some of these ubiquitination events may involve K6-linked Ub chains.

Many E3 ligases autoubiquitinate, and HECT E3s are known to assemble atypical Ub chains (Kristariyanto et al., 2015; Michel et al., 2015). We therefore assessed which chain types HUWE1 generated *in vitro*. The recombinant HUWE1 HECT domain assembled free and conjugated Ub chains, and close inspection of the assembly patterns comparing Ub WT with Ub K6R indicated assembly of K6 linkages (Figure 7B). This was confirmed by western blotting with the K6 affimer (Figure S7C), as well as by AQUA MS analysis. The latter, performed on the generated diUb, revealed that HUWE1 assembles K11 linkages (37%), K48 linkages (33%), K6 linkages (26%), and a small amount of K63 linkages (4%; Figure 7C), and similar results were obtained when the whole assembly reaction was analyzed (Figure S7D). To test whether full-length HUWE1 also assembles K6 chains *in vivo*, we analyzed the linkage composition of total cellular Ub chains in HUWE1$^{-/-}$ HeLa and parental control cell lines (Choe et al., 2016). Surprisingly, K6 chains were significantly less abundant in HUWE1$^{-/-}$ cell lines, showing a decrease of ~75% (Figure 7D). The only other chain type that changed significantly was K11, showing a decrease of ~18%. These results are consistent with a previous SILAC-based proteomic study aimed at identifying potential HUWE1 substrates, using a HEK293 cell line with doxycycline-inducible, short hairpin RNA (shRNA)-mediated knockdown of HUWE1 (Thompson et al., 2014). Extraction of Ub linkage data from the latter analysis confirmed the significant decrease in K6 chains upon knockdown of HUWE1 (Figure S7E). This was further substantiated in an inducible, shRNA-mediated HUWE1 knockdown system established in the colon cancer cell line Ls174T (Peter et al., 2014). TUBE

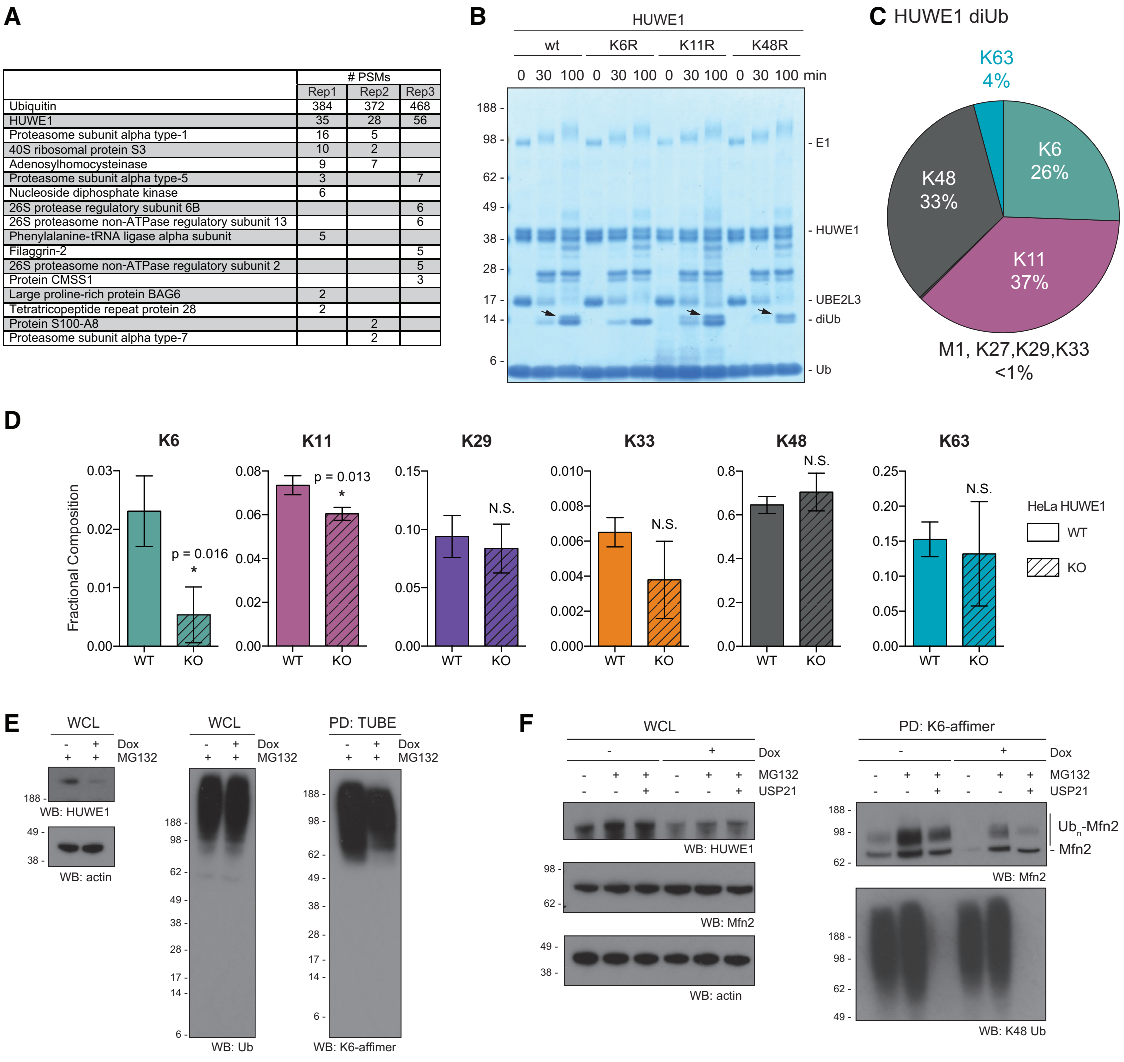

	# PSMs		
	Rep1	Rep2	Rep3
Ubiquitin	384	372	468
HUWE1	35	28	56
Proteasome subunit alpha type-1	16	5	
40S ribosomal protein S3	10	2	
Adenosylhomocysteinase	9	7	
Proteasome subunit alpha type-5	3		7
Nucleoside diphosphate kinase	6		
26S protease regulatory subunit 6B			6
26S proteasome non-ATPase regulatory subunit 13			6
Phenylalanine-tRNA ligase alpha subunit	5		
Filaggrin-2			5
26S proteasome non-ATPase regulatory subunit 2			5
Protein CMSS1			3
Large proline-rich protein BAG6	2		
Tetratricopeptide repeat protein 28	2		
Protein S100-A8		2	
Proteasome subunit alpha type-7		2	

Figure 7. HUWE1 Assembles K6 Chains *In Vitro* and *In Vivo*

(A) Table summarizing proteins identified with the corresponding number peptide-spectrum matches (PSMs) in three replicates of DUB-treated K6 affimer pull-downs.

(B) *In vitro* assembly reaction of the HECT E3 HUWE1 with Ub WT, Ub K6R, Ub K11R, and Ub K48R on Coomassie. Arrows indicate K6 diUb.

(C) Linkage composition of HUWE1-generated diUb after 1 hr as determined by AQUA MS.

(D) AQUA-derived total cellular chain composition of HUWE1$^{-/-}$ HeLa and parental cells after TUBE-based enrichment. Error bars indicate mean ± SD from n = 3. *$p < 0.05$, according to a two-tailed Student's t test. N.S., not significant.

(E) TUBE-PD from a doxycycline-inducible HUWE1 shRNA Ls174T cell line blotted with the K6-specific affimer, with input controls for actin, total Ub, and HUWE1.

(F) K6 affimer pull-down in doxycycline-inducible HUWE1 shRNA Ls174T cells blotted against Mfn2. Cells were left untreated or treated with 10 μg/mL MG132 for 4 hr and/or 1 μg/mL doxycycline for 72 hr. Pull-downs were incubated with 250 nM USP21 as indicated, with a K48 blot to show completeness of the deubiquitination reaction. Input controls are shown for Mfn2, actin, and HUWE1.

See also Figure S7.

pull-downs were analyzed by western blotting with the K6 affimer, and HUWE1 knockdown led to a decrease in K6 chains, whereas overall ubiquitination remained seemingly unaltered (Figure 7E). Together, this confirms that endogenous, full-length HUWE1 assembles K6 linkages (alongside K11 and K48 linkages) and is a major source of K6-linked chains in resting cells.

We next investigated ubiquitination of endogenous Mfn2, a known substrate of HUWE1 (Leboucher et al., 2012; Senyilmaz et al., 2015). We performed K6 affimer pull-downs from inducible HUWE1 shRNA Ls174T cells to enrich K6 ubiquitinated proteins. Western blotting for Mfn2 in uninduced cells reveals a slower migrating, ubiquitinated Mfn2 species, which is more pronounced after MG132 treatment (Figure 7F). Knockdown of HUWE1 leads to a marked reduction of ubiquitinated Mfn2 in the K6 affimer pull-down, with or without proteasome inhibition (Figure 7F). This indicates that Mfn2 is indeed a HUWE1 substrate.

Interestingly, we found a further way to corroborate the presence of K6 chains on substrates. The highly active and

non-specific DUB USP21 (Ye et al., 2011) was unable to cleave K6 linkages when bound to the K6 affimer, but it remained active against other chain types (Figures S7F and S7G). Similarly, the K33/K11 affimer fully protected K33 diUb and partially protected K11-linked diUb from USP21 cleavage (Figure S7H). Incubation of the K6 affimer pull-down with USP21 cleaved non-K6 chains and hydrolyzed all K48 linkages (the second most abundant chain type in this pull-down) detected by a K48-specific antibody (Figure 7F). Importantly, USP21 treatment only slightly reduced the ubiquitinated Mfn2 signal, indicating that the affimer protected K6-modified Mfn2 from being deubiquitinated by USP21. This is the most direct evidence that HUWE1-mediated ubiquitination of Mfn2 involves K6-linked polyUb.

DISCUSSION

The past years have seen significant progress in the study of atypical Ub signals, yet some chain types, in particular K6, K27, K29, and K33, have remained poorly studied due to lack of tools. Here, we partially fill this gap by establishing linkage-specific affimers for K6 and K33/K11 linkages. The characterized affimers have comparable properties to available linkage-specific antibodies and are suitable for a broad range of applications.

The crystal structures of affimers bound to their cognate chain types revealed how the cystatin scaffold dimerizes to achieve linkage specificity. Moreover, we have been able to predict a cross-reactivity in a Ub-binding protein when we realized that the K33 diUb conformation adopted in the affimer complex is similar to a conformation that K11 diUb can adopt (Bremm et al., 2010; Matsumoto et al., 2010). This insight supports the notion that the many distinct polyUb structures reported may each be individually meaningful and functional.

Whereas these affimers exhibit $\sim 10^6$-fold linkage specificity on the level of diUb, it is important to include appropriate controls when using these reagents in a cellular context to minimize off-target binding at the concentrations used in the particular assay. It should be established that global ubiquitination is unaffected (for example, after a stimulus) while there is a differential signal for the chain type in question. Also, DUB treatments should be used with caution—depending on where the Lys6 linkage is located (e.g., in distal parts of a mixed or branched chain), the signal for a K6-modified protein may inadvertently be lost. Ideal controls could include an ubiquitin replacement strategy (Ordureau et al., 2015; Xu et al., 2009), although, e.g., K6R mutation affects assembly of other chain types, such as K11 chains (Boname et al., 2010).

Further, we here identify three human E3 ligases, RNF144A, RNF144B, and HUWE1, that assemble K6/K11/K48 (and some K63) linkages. Parkin is a fourth ligase to assemble this linkage combination (Durcan et al., 2014; Ordureau et al., 2014). Our finding that loss of HUWE1 globally reduces the levels of K6 linkages indicates that we have identified a major ligase for this chain type. This is reminiscent of M1-linked chains, global levels of which are regulated by the RBR E3 ligase HOIP and the DUB OTULIN (Elliott, 2016). RBR ligases, such as Parkin, undergo sophisticated activation mechanisms and may only be active in particular cellular context or after certain stimulations (Swatek and Komander, 2016). Hence, like M1-linked chains, K6 linkages could be used as a precision signal in highly regulated contexts.

HUWE1 has been implicated in the degradation of numerous short-lived proteins, and its ability to assemble K6-/K11-/K48-linked polymers indicates that this chain configuration may be a powerful degradation tag. K11/K48 branched chains are an efficient proteasomal degradation signal (Meyer and Rape, 2014). Recent work on proteasomal Ub receptors has found a K6/K48 preference in Rpn1 (Shi et al., 2016). Also, the structural requirement for *exo* cleavage of K6 polyUb by USP DUBs (Mevissen and Komander, 2017) would not allow K6 chains to be cleaved en bloc by USP14 as recently proposed (Lee et al., 2016), which may extend residence time and degradation efficiency of K6-modified substrates at the proteasome. Indeed, K6 linkages accumulate quickly after proteasomal inhibition (Dammer et al., 2011). Nonetheless, HUWE1 is involved in many other contexts (e.g., DDR or mitophagy) where K6-linked chains could serve other roles, such as adaptor functions. The tools and protocols presented here to study K6- (and also K33-) linked Ub chains will enable further functional analysis of these unstudied cellular signals and will lead to a better understanding of the Ub code.

STAR★METHODS

Detailed methods are provided in the online version of this paper and include the following:

- KEY RESOURCES TABLE
- CONTACT FOR REAGENT AND RESOURCE SHARING
- EXPERIMENTAL MODELS AND SUBJECT DETAILS
- METHOD DETAILS
  - Molecular Biology
  - Protein Expression and Purification
  - Western Blotting
  - Isothermal Titration Calorimetry and Surface Plasmon Resonance
  - Fluorescence Polarization and Microscale Thermophoresis
  - Crystallization, Data Collection, and Refinement
  - SEC-MALS
  - Small-Scale Ub Assembly and Disassembly Reactions
  - Ub Chain Composition Analysis (AQUA)
  - Generation of NleL Cell Lines
  - TUBE Pull-Downs
  - Confocal Fluorescence Microscopy
  - Affimer Pull-Downs
  - Shotgun Proteomics
- QUANTITATION AND STATISTICAL ANALYSES
- DATA AND SOFTWARE AVAILABILITY

SUPPLEMENTAL INFORMATION

Supplemental Information includes seven figures and can be found with this article online at http://dx.doi.org/10.1016/j.molcel.2017.08.020.

AUTHOR CONTRIBUTIONS

M.A.M. performed all experiments with help from K.N.S. for mass spectrometry analyses. M.K.H. generated the inducible NleL cell line. M.A.M. and

D.K. conceived the project and wrote the manuscript with input from all authors.

ACKNOWLEDGMENTS

We thank Avacta for initial reagents and discussions on the project, Paul R. Elliott, Malte Gersch, and other members of the D.K. lab for reagents and advice, and beam line staff at ESRF and DLS for their assistance. We thank G.L. Moldovan (Pennsylvania State University) for HUWE1$^{-/-}$ and parental cells and M. Eilers (University of Wuerzburg) for dox-inducible Ls174T HUWE1 shRNA cell lines. Access to DLS was supported in part by the EU FP7 infrastructure grant BIOSTRUCT-X (contract no. 283570). Work in the D.K. lab is funded by the Medical Research Council (U105192732), the European Research Council (309756), the Michael J. Fox Foundation, and the Lister Institute for Preventive Medicine. M.A.M. was supported by a fellowship of the Boehringer Ingelheim Fonds and a Doc.Mobility fellowship of the Swiss National Science Foundation (P1SKP3_171785).

Received: April 6, 2017
Revised: July 13, 2017
Accepted: August 22, 2017
Published: September 21, 2017

REFERENCES

Adams, P.D., Afonine, P.V., Bunkóczi, G., Chen, V.B., Echols, N., Headd, J.J., Hung, L.-W., Jain, S., Kapral, G.J., Grosse Kunstleve, R.W., et al. (2011). The Phenix software for automated determination of macromolecular structures. Methods *55*, 94–106.

Bingol, B., and Sheng, M. (2016). Mechanisms of mitophagy: PINK1, Parkin, USP30 and beyond. Free Radic. Biol. Med. *100*, 210–222.

Boname, J.M., Thomas, M., Stagg, H.R., Xu, P., Peng, J., and Lehner, P.J. (2010). Efficient internalization of MHC I requires lysine-11 and lysine-63 mixed linkage polyubiquitin chains. Traffic *11*, 210–220.

Bremm, A., Freund, S.M.V., and Komander, D. (2010). Lys11-linked ubiquitin chains adopt compact conformations and are preferentially hydrolyzed by the deubiquitinase Cezanne. Nat. Struct. Mol. Biol. *17*, 939–947.

Choe, K.N., Nicolae, C.M., Constantin, D., Imamura Kawasawa, Y., Delgado-Diaz, M.R., De, S., Freire, R., Smits, V.A., and Moldovan, G.L. (2016). HUWE1 interacts with PCNA to alleviate replication stress. EMBO Rep. *17*, 874–886.

Clague, M.J., Barsukov, I., Coulson, J.M., Liu, H., Rigden, D.J., and Urbé, S. (2013). Deubiquitylases from genes to organism. Physiol. Rev. *93*, 1289–1315.

Cunningham, C.N., Baughman, J.M., Phu, L., Tea, J.S., Yu, C., Coons, M., Kirkpatrick, D.S., Bingol, B., and Corn, J.E. (2015). USP30 and parkin homeostatically regulate atypical ubiquitin chains on mitochondria. Nat. Cell Biol. *17*, 160–169.

Dammer, E.B., Na, C.H., Xu, P., Seyfried, N.T., Duong, D.M., Cheng, D., Gearing, M., Rees, H., Lah, J.J., Levey, A.I., et al. (2011). Polyubiquitin linkage profiles in three models of proteolytic stress suggest the etiology of Alzheimer disease. J. Biol. Chem. *286*, 10457–10465.

Durcan, T.M., Tang, M.Y., Pérusse, J.R., Dashti, E.A., Aguileta, M.A., McLelland, G.-L., Gros, P., Shaler, T.A., Faubert, D., Coulombe, B., and Fon, E.A. (2014). USP8 regulates mitophagy by removing K6-linked ubiquitin conjugates from parkin. EMBO J. *33*, 2473–2491.

Elia, A.E.H., Boardman, A.P., Wang, D.C., Huttlin, E.L., Everley, R.A., Dephoure, N., Zhou, C., Koren, I., Gygi, S.P., and Elledge, S.J. (2015). Quantitative proteomic atlas of ubiquitination and acetylation in the DNA damage response. Mol. Cell *59*, 867–881.

Elliott, P.R. (2016). Molecular basis for specificity of the Met1-linked polyubiquitin signal. Biochem. Soc. Trans. *44*, 1581–1602.

Emsley, P., Lohkamp, B., Scott, W.G., and Cowtan, K. (2010). Features and development of Coot. Acta Crystallogr. D Biol. Crystallogr. *66*, 486–501.

Evans, P.R., and Murshudov, G.N. (2013). How good are my data and what is the resolution? Acta Crystallogr. D Biol. Crystallogr. *69*, 1204–1214.

Friez, M.J., Brooks, S.S., Stevenson, R.E., Field, M., Basehore, M.J., Adès, L.C., Sebold, C., McGee, S., Saxon, S., Skinner, C., et al. (2016). HUWE1 mutations in Juberg-Marsidi and Brooks syndromes: the results of an X-chromosome exome sequencing study. BMJ Open *6*, e009537.

Gersch, M., Gladkova, C., Schubert, A., Michel, M.A., Maslen, S., and Komander, D. (2017). Mechanism and regulation of the Lys6-selective deubiquitinase USP30. Nat. Struct. Mol. Biol. http://dx.doi.org/10.1038/nsmb.3475.

Hjerpe, R., Aillet, F., Lopitz-Otsoa, F., Lang, V., England, P., and Rodriguez, M.S. (2009). Efficient protection and isolation of ubiquitylated proteins using tandem ubiquitin-binding entities. EMBO Rep. *10*, 1250–1258.

Hospenthal, M.K., Freund, S.M.V., and Komander, D. (2013). Assembly, analysis and architecture of atypical ubiquitin chains. Nat. Struct. Mol. Biol. *20*, 555–565.

Husnjak, K., and Dikic, I. (2012). Ubiquitin-binding proteins: decoders of ubiquitin-mediated cellular functions. Annu. Rev. Biochem. *81*, 291–322.

Janowski, R., Kozak, M., Jankowska, E., Grzonka, Z., Grubb, A., Abrahamson, M., and Jaskolski, M. (2001). Human cystatin C, an amyloidogenic protein, dimerizes through three-dimensional domain swapping. Nat. Struct. Biol. *8*, 316–320.

Kabsch, W. (2010). XDS. Acta Crystallogr. D Biol. Crystallogr. *66*, 125–132.

Kaiser, S.E., Riley, B.E., Shaler, T.A., Trevino, R.S., Becker, C.H., Schulman, H., and Kopito, R.R. (2011). Protein standard absolute quantification (PSAQ) method for the measurement of cellular ubiquitin pools. Nat. Methods *8*, 691–696.

Komander, D., and Rape, M. (2012). The ubiquitin code. Annu. Rev. Biochem. *81*, 203–229.

Kristariyanto, Y.A., Choi, S.-Y., Rehman, S.A.A., Ritorto, M.S., Campbell, D.G., Morrice, N.A., Toth, R., and Kulathu, Y. (2015). Assembly and structure of Lys33-linked polyubiquitin reveals distinct conformations. Biochem. J. *467*, 345–352.

Leboucher, G.P., Tsai, Y.C., Yang, M., Shaw, K.C., Zhou, M., Veenstra, T.D., Glickman, M.H., and Weissman, A.M. (2012). Stress-induced phosphorylation and proteasomal degradation of mitofusin 2 facilitates mitochondrial fragmentation and apoptosis. Mol. Cell *47*, 547–557.

Lee, B.H., Lu, Y., Prado, M.A., Shi, Y., Tian, G., Sun, S., Elsasser, S., Gygi, S.P., King, R.W., and Finley, D. (2016). USP14 deubiquitinates proteasome-bound substrates that are ubiquitinated at multiple sites. Nature *532*, 398–401.

MacLean, B., Tomazela, D.M., Shulman, N., Chambers, M., Finney, G.L., Frewen, B., Kern, R., Tabb, D.L., Liebler, D.C., and MacCoss, M.J. (2010). Skyline: an open source document editor for creating and analyzing targeted proteomics experiments. Bioinformatics *26*, 966–968.

Matsumoto, M.L., Wickliffe, K.E., Dong, K.C., Yu, C., Bosanac, I., Bustos, D., Phu, L., Kirkpatrick, D.S., Hymowitz, S.G., Rape, M., et al. (2010). K11-linked polyubiquitination in cell cycle control revealed by a K11 linkage-specific antibody. Mol. Cell *39*, 477–484.

Matsumoto, M.L., Dong, K.C., Yu, C., Phu, L., Gao, X., Hannoush, R.N., Hymowitz, S.G., Kirkpatrick, D.S., Dixit, V.M., and Kelley, R.F. (2012). Engineering and structural characterization of a linear polyubiquitin-specific antibody. J. Mol. Biol. *418*, 134–144.

McCoy, A.J., Grosse-Kunstleve, R.W., Adams, P.D., Winn, M.D., Storoni, L.C., and Read, R.J. (2007). Phaser crystallographic software. J. Appl. Cryst. *40*, 658–674.

Mevissen, T.E.T., and Komander, D. (2017). Mechanisms of deubiquitinase specificity and regulation. Annu. Rev. Biochem. *86*, 159–192.

Meyer, H.-J., and Rape, M. (2014). Enhanced protein degradation by branched ubiquitin chains. Cell *157*, 910–921.

Michel, M.A., Elliott, P.R., Swatek, K.N., Simicek, M., Pruneda, J.N., Wagstaff, J.L., Freund, S.M.V., and Komander, D. (2015). Assembly and specific recognition of k29- and k33-linked polyubiquitin. Mol. Cell *58*, 95–109.

Morris, J.R., and Solomon, E. (2004). BRCA1: BARD1 induces the formation of conjugated ubiquitin structures, dependent on K6 of ubiquitin, in cells during DNA replication and repair. Hum. Mol. Genet. *13*, 807–817.

Myant, K.B., Cammareri, P., Hodder, M.C., Wills, J., Von Kriegsheim, A., Győrffy, B., Rashid, M., Polo, S., Maspero, E., Vaughan, L., et al. (2017). HUWE1 is a critical colonic tumour suppressor gene that prevents MYC signalling, DNA damage accumulation and tumour initiation. EMBO Mol. Med. *9*, 181–197.

Narendra, D., Kane, L.A., Hauser, D.N., Fearnley, I.M., and Youle, R.J. (2010). p62/SQSTM1 is required for Parkin-induced mitochondrial clustering but not mitophagy; VDAC1 is dispensable for both. Autophagy *6*, 1090–1106.

Newton, K., Matsumoto, M.L., Wertz, I.E., Kirkpatrick, D.S., Lill, J.R., Tan, J., Dugger, D., Gordon, N., Sidhu, S.S., Fellouse, F.A., et al. (2008). Ubiquitin chain editing revealed by polyubiquitin linkage-specific antibodies. Cell *134*, 668–678.

Ordureau, A., Sarraf, S.A., Duda, D.M., Heo, J.-M., Jedrychowski, M.P., Sviderskiy, V.O., Olszewski, J.L., Koerber, J.T., Xie, T., Beausoleil, S.A., et al. (2014). Quantitative proteomics reveal a feedforward mechanism for mitochondrial PARKIN translocation and ubiquitin chain synthesis. Mol. Cell *56*, 360–375.

Ordureau, A., Heo, J.-M., Duda, D.M., Paulo, J.A., Olszewski, J.L., Yanishevski, D., Rinehart, J., Schulman, B.A., and Harper, J.W. (2015). Defining roles of PARKIN and ubiquitin phosphorylation by PINK1 in mitochondrial quality control using a ubiquitin replacement strategy. Proc. Natl. Acad. Sci. USA *112*, 6637–6642.

Pandya, R.K., Partridge, J.R., Love, K.R., Schwartz, T.U., and Ploegh, H.L. (2010). A structural element within the HUWE1 HECT domain modulates self-ubiquitination and substrate ubiquitination activities. J. Biol. Chem. *285*, 5664–5673.

Parsons, J.L., Tait, P.S., Finch, D., Dianova, I.I., Edelmann, M.J., Khoronenkova, S.V., Kessler, B.M., Sharma, R.A., McKenna, W.G., and Dianov, G.L. (2009). Ubiquitin ligase ARF-BP1/Mule modulates base excision repair. EMBO J. *28*, 3207–3215.

Peng, J., Schwartz, D., Elias, J.E., Thoreen, C.C., Cheng, D., Marsischky, G., Roelofs, J., Finley, D., and Gygi, S.P. (2003). A proteomics approach to understanding protein ubiquitination. Nat. Biotechnol. *21*, 921–926.

Peter, S., Bultinck, J., Myant, K., Jaenicke, L.A., Walz, S., Müller, J., Gmachl, M., Treu, M., Boehmelt, G., Ade, C.P., et al. (2014). Tumor cell-specific inhibition of MYC function using small molecule inhibitors of the HUWE1 ubiquitin ligase. EMBO Mol. Med. *6*, 1525–1541.

Sarraf, S.A., Raman, M., Guarani-Pereira, V., Sowa, M.E., Huttlin, E.L., Gygi, S.P., and Harper, J.W. (2013). Landscape of the PARKIN-dependent ubiquitylome in response to mitochondrial depolarization. Nature *496*, 372–376.

Scheffner, M., and Kumar, S. (2014). Mammalian HECT ubiquitin-protein ligases: biological and pathophysiological aspects. Biochim. Biophys. Acta *1843*, 61–74.

Senyilmaz, D., Virtue, S., Xu, X., Tan, C.Y., Griffin, J.L., Miller, A.K., Vidal-Puig, A., and Teleman, A.A. (2015). Regulation of mitochondrial morphology and function by stearoylation of TFR1. Nature *525*, 124–128.

Shi, Y., Chen, X., Elsasser, S., Stocks, B.B., Tian, G., Lee, B.-H., Shi, Y., Zhang, N., de Poot, S.A., Tuebing, F., et al. (2016). Rpn1 provides adjacent receptor sites for substrate binding and deubiquitination by the proteasome. Science *351*, 831.

Sung, M.-K., Porras-Yakushi, T.R., Reitsma, J.M., Huber, F.M., Sweredoski, M.J., Hoelz, A., Hess, S., and Deshaies, R.J. (2016). A conserved quality-control pathway that mediates degradation of unassembled ribosomal proteins. eLife *5*, 3429.

Swatek, K.N., and Komander, D. (2016). Ubiquitin modifications. Cell Res. *26*, 399–422.

Tarantino, N., Tinevez, J.-Y., Crowell, E.F., Boisson, B., Henriques, R., Mhlanga, M., Agou, F., Israël, A., and Laplantine, E. (2014). TNF and IL-1 exhibit distinct ubiquitin requirements for inducing NEMO-IKK supramolecular structures. J. Cell Biol. *204*, 231–245.

Thompson, J.W., Nagel, J., Hoving, S., Gerrits, B., Bauer, A., Thomas, J.R., Kirschner, M.W., Schirle, M., and Luchansky, S.J. (2014). Quantitative Lys-ϵ-Gly-Gly (diGly) proteomics coupled with inducible RNAi reveals ubiquitin-mediated proteolysis of DNA damage-inducible transcript 4 (DDIT4) by the E3 ligase HUWE1. J. Biol. Chem. *289*, 28942–28955.

Tiede, C., Tang, A.A.S., Deacon, S.E., Mandal, U., Nettleship, J.E., Owen, R.L., George, S.E., Harrison, D.J., Owens, R.J., Tomlinson, D.C., and McPherson, M.J. (2014). Adhiron: a stable and versatile peptide display scaffold for molecular recognition applications. Protein Eng. Des. Sel. *27*, 145–155.

Tiede, C., Bedford, R., Heseltine, S.J., Smith, G., Wijetunga, I., Ross, R., AlQallaf, D., Roberts, A.P., Balls, A., Curd, A., et al. (2017). Affimer proteins are versatile and renewable affinity reagents. eLife *6*, 515.

Virdee, S., Ye, Y., Nguyen, D.P., Komander, D., and Chin, J.W. (2010). Engineered diubiquitin synthesis reveals Lys29-isopeptide specificity of an OTU deubiquitinase. Nat. Chem. Biol. *6*, 750–757.

Wauer, T., Swatek, K.N., Wagstaff, J.L., Gladkova, C., Pruneda, J.N., Michel, M.A., Gersch, M., Johnson, C.M., Freund, S.M.V., and Komander, D. (2015). Ubiquitin Ser65 phosphorylation affects ubiquitin structure, chain assembly and hydrolysis. EMBO J. *34*, 307–325.

Wenzel, D.M., Lissounov, A., Brzovic, P.S., and Klevit, R.E. (2011). UBCH7 reactivity profile reveals parkin and HHARI to be RING/HECT hybrids. Nature *474*, 105–108.

Wu-Baer, F., Lagrazon, K., Yuan, W., and Baer, R. (2003). The BRCA1/BARD1 heterodimer assembles polyubiquitin chains through an unconventional linkage involving lysine residue K6 of ubiquitin. J. Biol. Chem. *278*, 34743–34746.

Xu, M., Skaug, B., Zeng, W., and Chen, Z.J. (2009). A ubiquitin replacement strategy in human cells reveals distinct mechanisms of IKK activation by TNFalpha and IL-1beta. Mol. Cell *36*, 302–314.

Xu, Y., Anderson, D.E., and Ye, Y. (2016). The HECT domain ubiquitin ligase HUWE1 targets unassembled soluble proteins for degradation. Cell Discov. *2*, 16040.

Yau, R., and Rape, M. (2016). The increasing complexity of the ubiquitin code. Nat. Cell Biol. *18*, 579–586.

Ye, Y., and Rape, M. (2009). Building ubiquitin chains: E2 enzymes at work. Nat. Rev. Mol. Cell Biol. *10*, 755–764.

Ye, Y., Akutsu, M., Reyes-Turcu, F., Enchev, R.I., Wilkinson, K.D., and Komander, D. (2011). Polyubiquitin binding and cross-reactivity in the USP domain deubiquitinase USP21. EMBO Rep. *12*, 350–357.

Ye, Y., Blaser, G., Horrocks, M.H., Ruedas-Rama, M.J., Ibrahim, S., Zhukov, A.A., Orte, A., Klenerman, D., Jackson, S.E., and Komander, D. (2012). Ubiquitin chain conformation regulates recognition and activity of interacting proteins. Nature *492*, 266–270.

Zheng, N., and Shabek, N. (2017). Ubiquitin ligases: structure, function, and regulation. Annu. Rev. Biochem. *86*, 129–157.

Zhong, Q., Gao, W., Du, F., and Wang, X. (2005). Mule/ARF-BP1, a BH3-only E3 ubiquitin ligase, catalyzes the polyubiquitination of Mcl-1 and regulates apoptosis. Cell *121*, 1085–1095.

STAR★METHODS

KEY RESOURCES TABLE

REAGENT or RESOURCE	SOURCE	IDENTIFIER
Antibodies		
α-Actin (clone C4)	Millipore	MAB1501R; RRID: AB_2223041
α-HA (clone 3F10)	Roche	11867423001; RRID: AB_390918
α-Ub	Novus Biologicals	Ubi-1; RRID: AB_2238516
α-γH2Ax	Millipore	JBW301; RRID: AB_310795
α-HUWE1	Bethyl Laboratories	A300-486A; RRID: AB_2264590
α-Mfn2	Abcam	ab56889; RRID: AB_2142629
α-K48 Ub	Millipore	Apu2; RRID: AB_1587578
α-K63 Ub	Millipore	Apu3; RRID: AB_1587580
α-TOM20	Santa Cruz Biotechnology	FL-145/sc-11415; RRID: AB_2207533
FK2	Millipore	FK2; RRID: AB_612093
α-NEMO	Santa Cruz Biotechnology	FL-419/sc-8330; RRID: AB_2124846
HRP-conjugated Streptavidin	CST	3999; RRID: AB_10830897
HRP-conjugated sheep α-mouse	GE Life Sciences	NA931; RRID: AB_772210
HRP-conjugated donkey α-rabbit	GE Life Sciences	NA934; RRID: AB_772206
Alexa 647-conjugated donkey α-mouse	Invitrogen	A31571; RRID: AB_162542
Alexa 594-conjugated donkey α-rabbit	Invitrogen	A21207; RRID: AB_141637
Chemicals, Peptides, and Recombinant Proteins		
K27 diUb	UbiQ	UbiQ-015
TNF-α	Invitrogen	PHC3016
Deposited Data		
K6-affimer:K6 diUb structure	This paper	PDB: 5OHL
K33-affimer:K33 diUb structure (*H* 3)	This paper	PDB: 5OHV
K33-affimer:K33 diUb structure (*P* 2_1)	This paper	PDB: 5OHM
Experimental Models: Cell Lines		
HEK293	N/A	N/A
Ls174T with HUWE1 shRNA	Peter et al., 2014	# 2
HeLa parental	Choe et al., 2016	N/A
HeLa HUWE1 KO	Choe et al., 2016	N/A
T-REx 293 HA-NleL	This study	N/A
Flp-In T-Rex HeLa Parkin wild-type	Ordureau et al., 2014	N/A
Flp-In T-Rex HeLa Parkin C431S	Ordureau et al., 2014	N/A
Software and Algorithms		
MicroCal ITC Origin Analysis software	Malvern	N/A
Skyline	MacLean et al., 2010	N/A
Proteome Discoverer	Thermo Scientific	N/A
Other		
Protease inhibitor cocktail	Roche	11697498001
MG132	Sigma-Aldrich	C2211
CCCP	Sigma-Aldrich	C2759
EZ-Link Maleimide-PEG2-Biotin	Thermo Scientific	21901BID
KOD HotStart Polymerase	Novagen	71086
In-Fusion HD Cloning Kit	Clontech	638918
DNase I	Sigma-Aldrich	DN25

(*Continued on next page*)

Continued		
REAGENT or RESOURCE	SOURCE	IDENTIFIER
Lysozyme	Sigma-Aldrich	L6876
TALON resin	Clontech	635504
Glutathione resin	Amintra	AGS0100
Benzonase	Novagen	70664
NuPAGE 4%–12% Bis-Tris Gel	Invitrogen	NP0322
Trans-Blot Turbo Nitrocellulose Membrane	Bio-Rad	1704158
Alexa 488 C5 maleimide	Thermo Scientific	A10254
NT-647 maleimide	NanoTemper	MO-L004
Dynabeads M-280 Streptavidin	Invitrogen	11205D
Antimycin A	Sigma	A8674
Oligomycin	Millipore	495455

CONTACT FOR REAGENT AND RESOURCE SHARING

Requests for further information or reagents should be directed to the lead contact and corresponding author, David Komander (dk@mrc-lmb.cam.ac.uk). Request for cell lines from other studies should be directed to the respective corresponding authors. Affimers can be obtained from Avacta (https://www.avacta.com).

EXPERIMENTAL MODELS AND SUBJECT DETAILS

Ls174T cells were grown in RPMI1640 + 10% FCS (Fetal Calf Serum) +Pen/Strep, all other cell lines were grown in DMEM + 10% FCS + Pen/Strep. Cells were cultured at 37°C in a humidified atmosphere with 5% CO_2.

METHOD DETAILS

Molecular Biology

DNA sequences were amplified using KOD HotStart DNA polymerase. RNF144A (aa 16-228) and RNF144B (aa 27-236) were cloned into pOPIN-K, which encodes a 3C-cleavable N-terminal His_6-GST-tag using the In-Fusion HD cloning kit. Similarly, HUWE1 (aa 3993-4374) was cloned into pOPIN-B which encodes an N-terminal, 3C-cleavable His_6-tag. Coding sequences for RNF144A and RNF144B are a gift from Christopher Sanderson (University of Liverpool) and HUWE1 from Mark Bycroft (MRC LMB). All constructs were verified by DNA sequencing.

Protein Expression and Purification

RNF144A and RNF144B and HUWE1 were expressed in Rosetta2 (DE3) pLacI cells. Cells were grown from overnight cultures in 2xTY medium, supplemented with 35 μg/mL chloramphenicol and 50 μg/mL kanamycin. The cultures were cooled to 18°C prior to induction with 200 μM IPTG and expressed overnight. Pellets were resuspended in binding buffer (50 mM Tris pH 8.5, 150 mM NaCl, 2 mM β-mercaptoethanol). Prior to lysis by sonication, cell suspensions were supplemented with DNaseI, lysozyme and protease inhibitor cocktail. Proteins were bound to TALON metal affinity resin and washed using binding buffer supplemented with 5 mM imidazole. His_6-GST-tags of pOPIN-B constructs were cleaved by addition of a His_6-tagged 3C protease to beads at 4°C overnight. Eluted proteins were purified to homogeneity using size exclusion chromatography (HiLoad 16/60 Superdex 75, GE Life Sciences) in SEC buffer (20 mM Tris pH 8.5, 150 mM NaCl, 2 mM DTT). Peak fractions were concentrated, flash-frozen in liquid nitrogen and stored at −80°C.

HUWE1 (Pandya et al., 2010), Tandem UBA repeat TUBE (Hjerpe et al., 2009), Ub and E1, E2 and E3 enzymes and DUBs (Michel et al., 2015) were purified as previously described. Except for K27 diUb (UbiQ), all Ub chains were produced enzymatically as previously described (Bremm et al., 2010; Hospenthal et al., 2013; Michel et al., 2015).

Affimers are available from Avacta Life Sciences (Wetherby, UK).

Western Blotting

For western blotting, biotinylated affimers were used. A single cysteine was introduced in the N terminus of the affimers to allow for site-specific labeling by Maleimide-PEG2-Biotin (the affimer scaffold does not contain any cysteines otherwise). 5 mg of affimer was labeled in 1 mL of labeling buffer (20 mM Tris pH 7.4, 150 mM NaCl, 2 mM TCEP) with 20-fold molar excess of biotin-maleimide for 4 hr at 4°C. The reaction was stopped with 20 mM β-mercaptoethanol. Excess biotin was removed using PD10 desalting columns (GE Life Sciences). Samples for blotting with the affimers were generally not boiled, as the affimers recognize folded Ub. After blotting, the

nitrocellulose membrane was blocked in PBST containing 5% milk and incubated overnight at 4°C in 5% milk containing 0.1 μg affimer/mL. After a short wash in PBST, the blot was incubated for 1 hr with Streptavidin-HRP. For more sensitive detection, the blot was instead incubated for 1 hr at room temperature with an anti-biotin antibody, which was then incubated with a secondary, HRP-conjugated antibody. The signal was detected using Amersham ECL Prime reagent. Dilutions for all other western blotting reagents were 1:1,000, apart for Mfn2 (1:800), HA and actin (1:5,000) and HRP-conjugated secondaries (1:10,000).

Isothermal Titration Calorimetry and Surface Plasmon Resonance

Isothermal titration calorimetry (ITC) experiments were carried out at 25°C on a MicroCal iTC200 instrument (GE Life Sciences). Monomeric affimers and diUbs were dialyzed against PBS buffer (18 mM Na_2HPO_4, 7 mM NaH_2PO_4 pH 7.2, 150 mM NaCl). Following a pre-injection of 0.5 μL of diUb, diUb (30 μM) was injected in 19 × 2 μL / 49 × 0.5 μL / 79 × 0.5 μL consecutive injections into the monomeric affimer sample (5 μM) in the cell at 120 s intervals. After removing the pre-injection data point, the resulting binding curves were fitted and binding constants calculated in MicroCal ITC Origin Analysis software (Malvern).

Surface plasmon resonance (SPR) experiments were performed on a Biacore 2000 (GE Life Sciences) as previously described (Michel et al., 2015). Briefly, CM5 chips (GE Life Sciences) were activated, and functionalized by diUb injection at 100 ng/μL until 2000 response units were reached. For qualitative kinetic measurements, the samples were buffer exchanged into SPR buffer (20 mM Tris pH 7.4, 150 mM NaCl), and injected at 10 μM for 60 s followed by 150 s dissociation in SPR buffer at 20°C. Data were plotted in Prism 6.

Fluorescence Polarization and Microscale Thermophoresis

Affimers were labeled site-specifically on a single cysteine with Alexa488-maleimide (for FP and MST measurements on the Monolith NT.115) or NT-647-maleimide (for MST measurements on the Monolith.NT115pico) according to the manufacturer's instructions. Free dye was removed and the protein buffer exchanged into MST buffer (20 mM Tris pH 7.4, 150 mM NaCl, 0.05% Tween-20). For FP assays, 500 pM of labeled affimer was incubated with varying concentrations of diUb for 3 hr before measurements were taken on a PheraStar plate reader (BMG Labtech), equipped with an optic module for detection of Alexa488 dye (λ_{ex} = 485 nm, λ_{em} = 520 nm) at 25°C. The polarization value of free, labeled affimer was determined by a spectrofluorometer and used for referencing raw data. For fitting, values were converted into anisotropy values and fitted with GraphPad Prism with the following single-site binding equation to account for ligand depletion:

$$y = P_{min} + (P_{max} - P_{min}) \frac{(L + K_d + x) - \sqrt{(-L - K_d - x)^2 - 4Lx}}{2L},$$

where x is the concentration of diUb titrated and L the concentration of labeled affimer.

Kinetic association data were fitted using a one-phase association model described by

$$y = P_{min} + (P_{max} - P_{min})\left(1 - e^{-kx}\right),$$

where x is time and k is the rate constant, and the half-time (τ) can be calculated by $\tau = \ln(2)/k$.

All MST measurements were performed on a Monolith NT.115 (NanoTemper) with 500 pM labeled affimer, except for binding assays involving dimeric affimers and their cognate diUb. These were performed on a Monolith NT.115Pico instrument to allow for lower concentration of fluorescently affimer used (50 pM) and therefore more precise binding assays. Measurements were performed in MST buffer at 100% LED power and 60% MST laser power. Curve fitting was performed on data derived from either thermophoresis or temperature jump, whichever was greater in amplitude. Data were fitted as described for FP. All assays were performed in at least n = 3.

Crystallization, Data Collection, and Refinement

For crystals of K6-affimer bound to K6 diUb, 1 molar equivalent of K6-affimer was mixed with 1.2 molar equivalents of K6 diUb at a concentration of 8 mg/mL. Crystals grew at room temperature from a 2:1 (v/v) ratio of protein to reservoir solution containing 32.5% PEG 2000 MME, 200 mM ammonium acetate and 100 mM Tris pH 8.5. For vitrification, crystals were cryoprotected by transfer to a solution of mother liquor containing 28% glycerol.

For crystallization of the K33-affimer bound to K33 diUb, 1 molar equivalent of K33-affimer was mixed with 1.2 molar equivalents of K33 diUb at a final concentration of 5.7 mg/mL. Crystals grew by vapor diffusion at 4°C by mixing an equal volume of protein with 21 % PEG 3350, 200 mM $LiSO_4$ and 100 mM sodium acetate pH 5.2. Prior to vitrification, crystals were cryoprotected in mother liquor containing 25% glycerol. The 3.8 Å K33-affimer:K33 diUb crystals in space group $P2_1$ were grown at room temperature in 1:1 ratio of protein and 20% PEG 3350 and 200 mM KSCN and cryoprotected in mother liquor containing 20% glycerol.

Diffraction data were collected at ESRF beamline ID23-2 and Diamond Light Source beamlines I04 and I04-1. Diffraction data were integrated using XDS (Kabsch, 2010) and scaled using AIMLESS (Evans and Murshudov, 2013). Structures of higher resolution than 3 Å were solved by molecular replacement using truncated versions of the Adhiron scaffold (PDB: 4N6U) and Ub (PDB: 1UBQ) as search models in PHASER (McCoy et al., 2007), whereas the 3.8 Å K33-affimer:K33 diUb structure was solved by molecular replacement using the higher resolution structure of the complex. Both crystal forms of the K33-affimer complex were multiple,

and diffraction patterns showed a second, weakly diffracting lattice, which resulted in high R_{merge} values. Iterative rounds of manual model building and computational refinement were performed using COOT (Emsley et al., 2010) and PHENIX (Adams et al., 2011), respectively. All structural figures were created in PyMOL (http://www.pymol.org). Final statistics can be found in Table 1.

SEC-MALS

SEC-MALS measurements were performed using a Wyatt Dawn Heleos-II angle light scattering instrument connected to a Wyatt Optilab rEX online refractive index detector. Samples were diluted in SPR buffer to 200 μM for affimer only samples and to 118 μM affimer: 57 μM diUb for other samples, respectively. 100 μL of the diluted samples were run at 0.5 mL/min on an analytical gel filtration column (Superdex 75 10/300 GL, GE Life Sciences), before passing through the light scattering and refractive index detectors in a standard SEC-MALS format. Protein concentration was determined from the excess refractive index based on 0.19 RI for 1 mg/mL, and combined with the observed scattered intensity at each point in the chromatograms to calculate absolute molecular mass using Wyatt's ASTRA analysis software.

Small-Scale Ub Assembly and Disassembly Reactions

Ub chains were assembled in a reaction containing 0.1 μM E1, 2.5 μM E2, 2.5 μM E3 and 30 μM Ub variant in assembly buffer (50 mM Tris pH 8.0, 10 mM $MgCl_2$, 0.6 mM DTT, 10 mM ATP, 5% glycerol) at 37°C for the indicated times. For DUB assays, 10 μM diUb was incubated with 250 nM USP21 in DUB buffer (50 mM Tris pH 7.5, 50 mM NaCl, 5 mM DTT) at 37°C in the presence or absence of 12 μM dimeric K6-affimer-GFP. Reactions were stopped by addition of 4x SDS loading dye.

Ub Chain Composition Analysis (AQUA)

Mass spectrometry analyses were performed as previously described (Michel et al., 2015; Wauer et al., 2015). Briefly, samples were resolved in a SDS-PAGE gel before being excised and diced into 1 mm^3 pieces. Samples were digested with trypsin for 16 hr at 37°C after which 400 fmoles of isotopically labeled standards corresponding to each ubiquitinated peptide was added to the digestion reaction. Peptides were extracted from the gel slices, lyophilized, and resuspended in reconstitution buffer (7.5% acetonitrile, 0.5% TFA, 0.01% H_2O_2). Samples were separated using a Dionex Ultimate 3000 HPLC system with an EASY-Spray column (C18, 3 μm, 100Å, 75 μm x 15 μm) and analyzed on a Q Exactive (Thermo Scientific) using a parallel reaction-monitoring assay. Transition ions for the heavy and light peptides were quantified using Skyline (MacLean et al., 2010).

Generation of NleL Cell Lines

To create T-REx 293 Ha-NleL cell lines, pcDNA4/TO/N-2xHA containing full-length, codon-optimized NleL was linearized using ScaI and transfected into T-REx 293 cells (Invitrogen). Cells were selected with 150 μg/mL zeocin and individual colonies were expanded and screened for NleL expression by western blotting with an HA-antibody. NleL expression was induced with 1 μg/mL doxycycline for 12 hr at 70% confluency and TUBE pull-downs were performed as described below.

TUBE Pull-Downs

Cells were grown to 80% confluency. One 10 cm^2 plate was lysed in 1 mL TUBE buffer (1% NP-40, 2 mM EDTA, 10 mM chloroacetamide, 100 μg/mL GST-tagged tandem UBA repeat TUBE (Hjerpe et al., 2009), protease inhibitor cocktail in PBS) with sonication. After clearing by centrifugation, the lysate was incubated on a spinning wheel overnight at 4°C. 40 μL of glutathione beads were added and incubated for 1 hr. Beads were washed 4 times with 1 mL of PBST.

Confocal Fluorescence Microscopy

Inducible HeLa Flp-In T-REx cells expressing wild-type or catalytically inactive (C431S) Parkin (a kind gift of A. Ordureau & W. Harper (Harvard)) were seeded onto coverslips and expression was induced the next day with 0.2 μg/ml doxycycline for 16 hr. Mitochondria were depolarized using a combination of 4 μM Antimycin A and 10 μM Oligomycin for 2 hr. For TNFα experiments, HeLa cells were grown on coverslips and treated for 12 min with 10 ng/ml TNFα, before saponin extraction according to Tarantino et al., 2014. Cells for both sets of experiments were fixed using 4% paraformaldehyde, blocked using blocking buffer (5% goat serum and 0.2% Triton X-100 in PBS) and incubated overnight at room temperature in blocking buffer containing Alexa488-labeled dimerized K6-affimer (0.25 μg/ml), rabbit α-TOM20 (1:100) and FK2 (1:500) antibodies. After three washes in PBS, slides were incubated for 1 hr at room temperature in blocking buffer containing anti-mouse Alexa647- and anti-rabbit Alexa594-labeled antibodies (1:1000 each). Coverslips were mounted onto slide using Prolong Diamond Antifade mounting medium, sealed with nail polish and stored at 4°C. Imaging was performed on a Zeiss 780 with 63x magnification.

Affimer Pull-Downs

HEK293 cells were grown to confluency and incubated for 1 hr with 10 μM MG132, scraped in lysis buffer (100 mM Tris pH 8.0, 50 mM NaCl, 0.5% NP-40, 10 mM chloroacetamide, protease inhibitor cocktail, 1 mM PMSF, 50 U/mL benzonase, 5 % glycerol) containing the indicated amounts of GFP-tagged dimeric K6-affimer. Cell lysates were cleared and incubated overnight at 4°C. 5-15 μL GFP-trap beads (Chromo-Tek) were added and incubated for 1 hr at 4°C. The beads were then washed 5 times in lysis buffer containing

150 mM NaCl, and 1x with lysis buffer containing 500 mM NaCl. For samples treated with DUBs, 1 μM of improved AMSH and OTUB1 (Michel et al., 2015) were added and incubated for 1 hr at room temperature.

For Mfn2 ubiquitination experiments, Ls174T cells expressing a doxycycline-inducible shRNA targeting HUWE1 (Peter et al., 2014) were grown to 50% confluency and shRNA expression was induced by 1 μg/mL doxycycline for 72 hr. The medium containing doxycycline was replaced every 24-48 hr. Prior to lysis, cells were treated for 4 hr with 10 μg/mL MG132, as indicated. Per condition, 2 × 15 cm^2 dishes were lysed in lysis buffer containing 5 μg GFP-K6-affimer/mL. After washing the beads, 250 nM USP21 was added to beads at 4°C for 1 hr, as indicated.

For pull-downs with the K33/K11-affimer, 200 nM diUb was incubated with 200 nM site-specifically biotinylated K33/K11-affimer overnight in PBS. 10 μL of Dynabeads M-280 Streptavidin was added per sample, incubate for 1 hr at 4°C and samples were washed 5 x with PBST.

Proteins were eluted by boiling the samples in 4X LDS sample buffer, resolved by SDS-PAGE, transferred onto a nitrocellulose membrane and blotted as described above.

Shotgun Proteomics

Peptides for discovery proteomics were separated identically to the Ub AQUA analysis. However, a Top10 analysis was performed. Precursor masses were screened using the following settings: mass range, 200-2000 m/z; resolution, 70,000, AGC target, 1E6; maximum ion trap time, 250 ms; scan-type, positive. Data-dependent settings include the following: resolution, 17,500, AGC target, 5E4; maximum ion trap time, 80 ms; isolation window, 2.0 m/z; collision energy, 28.0; data type, centroid; exclusion of unassigned charge states and masses with a charge state of 1. Dynamic exclusion enabled, 30 s. Raw files were searched and spectra assigned using SEQUEST against a human genome database (UniProt) in Proteome Discoverer (Thermo Scientific) with a false-discovery rate of 1%.

QUANTITATION AND STATISTICAL ANALYSES

Western blots were quantified in ImageJ by normalizing on the actin signal. All error bars are represented as mean ± standard deviation. Statistical significance was calculated using a two-tailed Student's t test and significance is denoted as followed: N.S not significant, * $p < 0.05$, *** $p < 0.001$.

DATA AND SOFTWARE AVAILABILITY

Crystal structures have been deposited on the Protein Databank (PDB) under the following accession codes: K6-affimer:K6 diUb (*P* 1, PDB: 5OHL), K33-affimer:K33 diUb (*H* 3, PDB: 5OHV), K33-affimer:K33 diUb ($P\ 2_1$, PDB: 5OHM).

Molecular Cell, Volume *68*

Supplemental Information

Ubiquitin Linkage-Specific Affimers Reveal Insights into K6-Linked Ubiquitin Signaling

Martin A. Michel, Kirby N. Swatek, Manuela K. Hospenthal, and David Komander

Figure S1

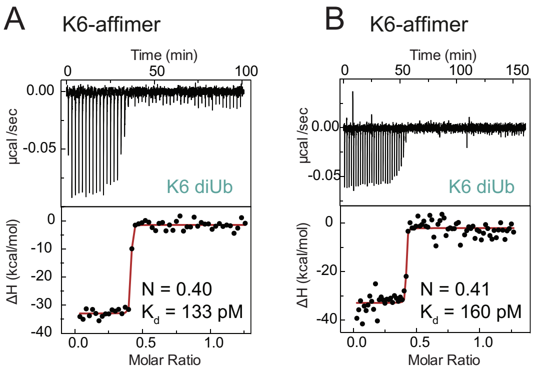

C

	K_d ± SEM (pM)	N ± SEM	ΔH ± SEM (kcal/mol)	ΔG (kcal/mol)
K33-affimer : K33 diUb	211 ± 280	0.44 ± 0.02	-16.2 ± 0.24	-24.6
K33-affimer : K11 diUb	7040 ± 4410	0.40 ± 0.07	-17.6 ± 0.70	-11.1
K6-affimer : K6 diUb	172 ± 579	0.46 ± 0.01	-33.3 ± 0.46	-13.3
K6-affimer : K6 diUb	133 ± 140	0.40 ± 0.02	-31.5 ± 0.46	-13.5
K6-affimer: K6 diUb	160 ± 233	0.41 ± 0.02	-30.8 ± 0.83	-13.4

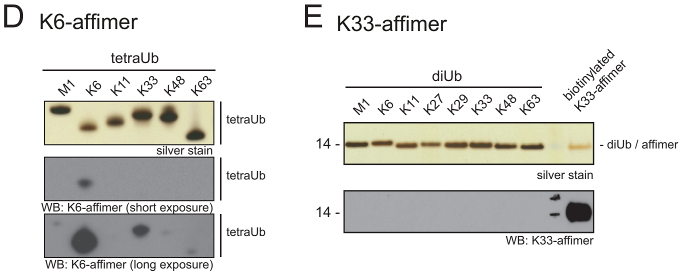

Supplementary Figure Legends

Figure S1 (related to Figure 1): Linkage-specificity of affimers

A-B) ITC curves of K6-affimer (5 µM, in cell) binding to K6 diUb (30 µM, in syringe) with A) 49 x 0.5 µL and B) 79 x 0.5 µL injections, respectively. Plots for raw heat (*top*) and derived isotherms (*bottom*) with fits (red) are shown. **C)** Table for best-fit parameters for ITC experiments from Figure 1 and S1. **D)** Western blot of the K6-affimer against differently linked tetraUb chains. **E)** Western blot with the biotinylated K33-affimer against differently linked diUb. Biotinylated K33-affimer was also loaded onto the gel as a technical control for detection with Streptavidin-HRP.

Figure S2

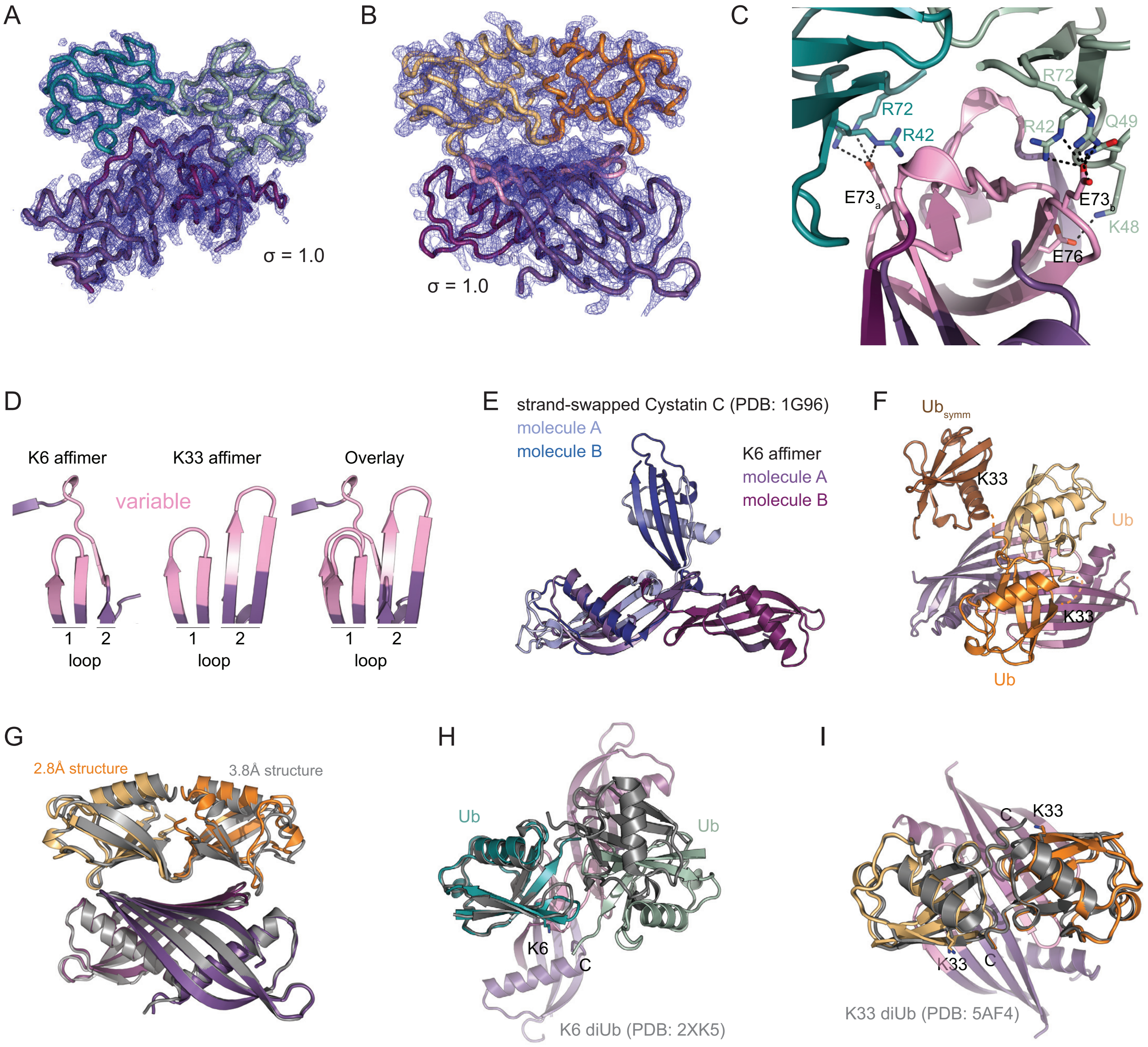
A
σ = 1.0
B
σ = 1.0
C
R72
R42
R72
R42
Q49
E73a
E73b
K48
E76
D
K6 affimer
K33 affimer
Overlay
variable
1
2
loop
1
2
loop
1
2
loop
E
strand-swapped Cystatin C (PDB: 1G96)
molecule A
molecule B
K6 affimer
molecule A
molecule B
F
Ubsymm
K33
Ub
K33
Ub
G
2.8Å structure
3.8Å structure
H
Ub
Ub
K6
C
K6 diUb (PDB: 2XK5)
I
C
K33
K33
C
K33 diUb (PDB: 5AF4)

Figure S2 (related to Figure 2): Structures of linkage-specific affimers with their cognate diUbs

A) Structure of K6-affimer bound to K6 diUb with corresponding 2 $|F_o|$ - $|F_c|$ electron density map, contoured at $\sigma = 1.0$. **B)** As in A, but for the K33-affimer:K33 diUb structure. **C)** Close up of charged interaction in the K6-affimer:K6 diUb structure, mediated by the variable region (pink) of the affimer. **D)** Variable loops (pink) of K6- and K33-affimers, and in overlay, showing the difference in secondary structure. **E)** Overlay of the strand-swapped K6-affimer (orange) with naturally occuring human cystatin C (blue, PDB: 1G96) that is also strand-swapped. Other naturally occuring cystatins engage in the strand swap in the same fashion as cystatin C, but the relative orientation of the two monomers differs. **F)** Symmetry-related molecules of the K33-affimer:K33 diUb structure showing how the bound chain can be extended. **G)** Overlay of the structure of the K33-affimer:K33 diUb in the *H3* space group (2.5Å) and the $P2_1$ space group (3.8Å, grey). **H)** Overlay of the K6-affimer:K6 diUb structure with previously solved K6 diUb structure (grey, PDB: 2XEW), superimposed on the distal Ub. **I)** As in E, but for the K33-affimer:K33 diUb complex and K33 diUb (grey, PDB: 5AF4), respectively.

Figure S3

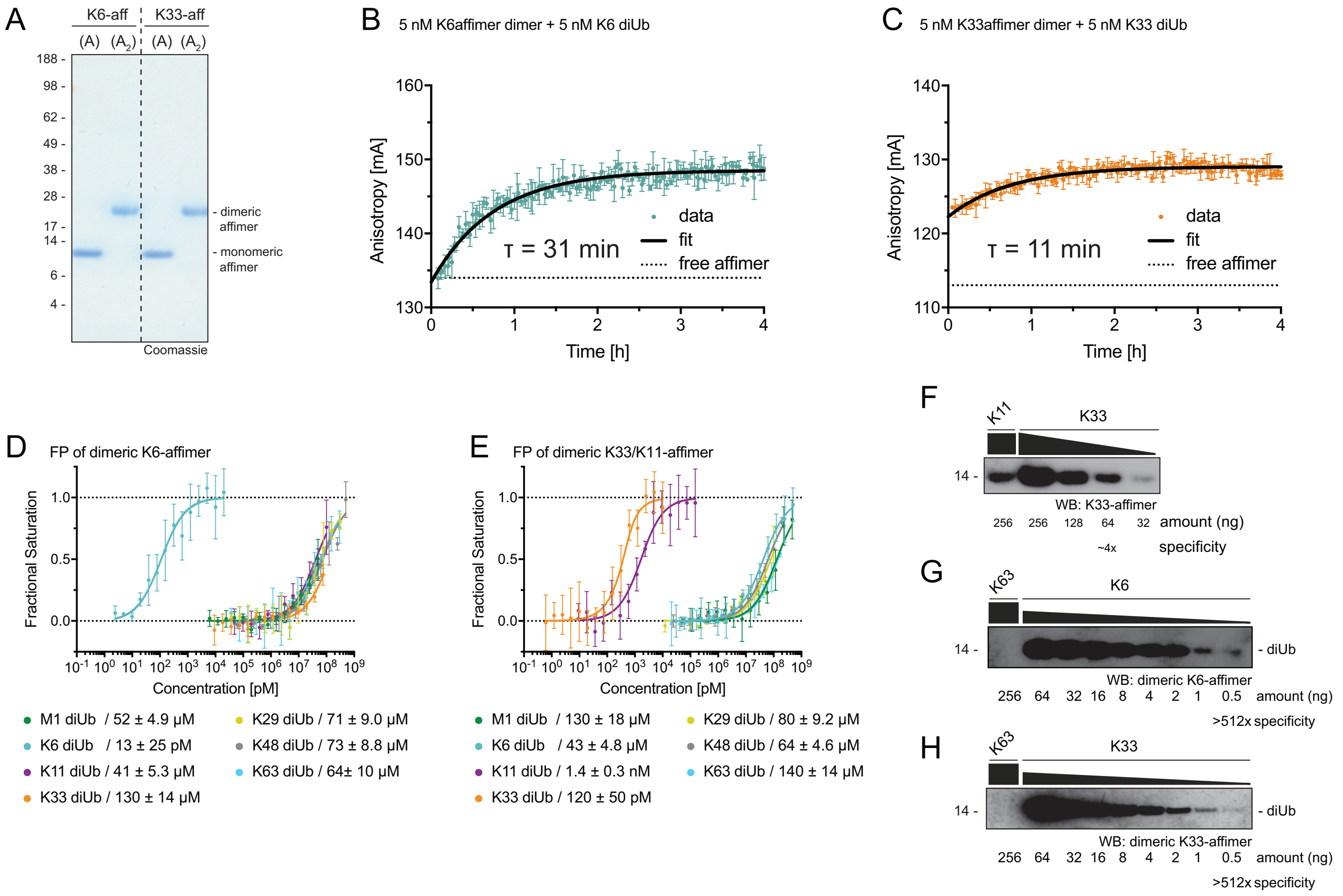
A
K6-aff
K33-aff
(A) (A2) (A) (A2)
188 -
98 -
62 -
49 -
38 -
28 -
17 -
14 -
6 -
4 -
- dimeric affimer
- monomeric affimer
Coomassie
B
5 nM K6affimer dimer + 5 nM K6 diUb
Anisotropy [mA]
τ = 31 min
data
fit
free affimer
Time [h]
C
5 nM K33affimer dimer + 5 nM K33 diUb
Anisotropy [mA]
τ = 11 min
data
fit
free affimer
Time [h]
D
FP of dimeric K6-affimer
Fractional Saturation
Concentration [pM]
M1 diUb / 52 ± 4.9 μM
K6 diUb / 13 ± 25 pM
K11 diUb / 41 ± 5.3 μM
K33 diUb / 130 ± 14 μM
K29 diUb / 71 ± 9.0 μM
K48 diUb / 73 ± 8.8 μM
K63 diUb / 64± 10 μM
E
FP of dimeric K33/K11-affimer
Fractional Saturation
Concentration [pM]
M1 diUb / 130 ± 18 μM
K6 diUb / 43 ± 4.8 μM
K11 diUb / 1.4 ± 0.3 nM
K33 diUb / 120 ± 50 pM
K29 diUb / 80 ± 9.2 μM
K48 diUb / 64 ± 4.6 μM
K63 diUb / 140 ± 14 μM
F
K11
K33
14 -
WB: K33-affimer
256 256 128 64 32 amount (ng)
~4x specificity
G
K63
K6
14 -
- diUb
WB: dimeric K6-affimer
256 64 32 16 8 4 2 1 0.5 amount (ng)
>512x specificity
H
K63
K33
14 -
- diUb
WB: dimeric K33-affimer
256 64 32 16 8 4 2 1 0.5 amount (ng)
>512x specificity

Figure S3 (related to Figure 3): Affimers dimerize to achieve linkage-specificity
A) Coomassie-stained gel of monomeric and dimerized versions of K6- and K33/K11-affimers, respectively. **B)** Fluorescence anisotropy time course with 5 nM K6-affimer and 5 nM K6 diUb. The dotted line indicates the anisotropy value for free K6-affimer and the data was fitted using a simple one-phase association model. **C)** As in B but for the K33/K11-affimer. **D)** Fluorescence polarization (FP) binding assay for the K6-affimer binding to differently linked diUb fitted with a one-site binding model accounting for ligand depletion. **E)** As in D, but for the K33/K11-affimer. **F)** Western blot using the dimerized K6-affimer using the indicated amounts of K6 diUb or K63 diUb .**G)** As in F, but probed with the dimerized K33/K11-affimer. **H)** Indicated amounts of K33 and K11 diUb were probed with the K33/K11-affimer by Western blotting. The specificity was estimated from relative signal strength and corresponding amounts loaded.

Figure S4

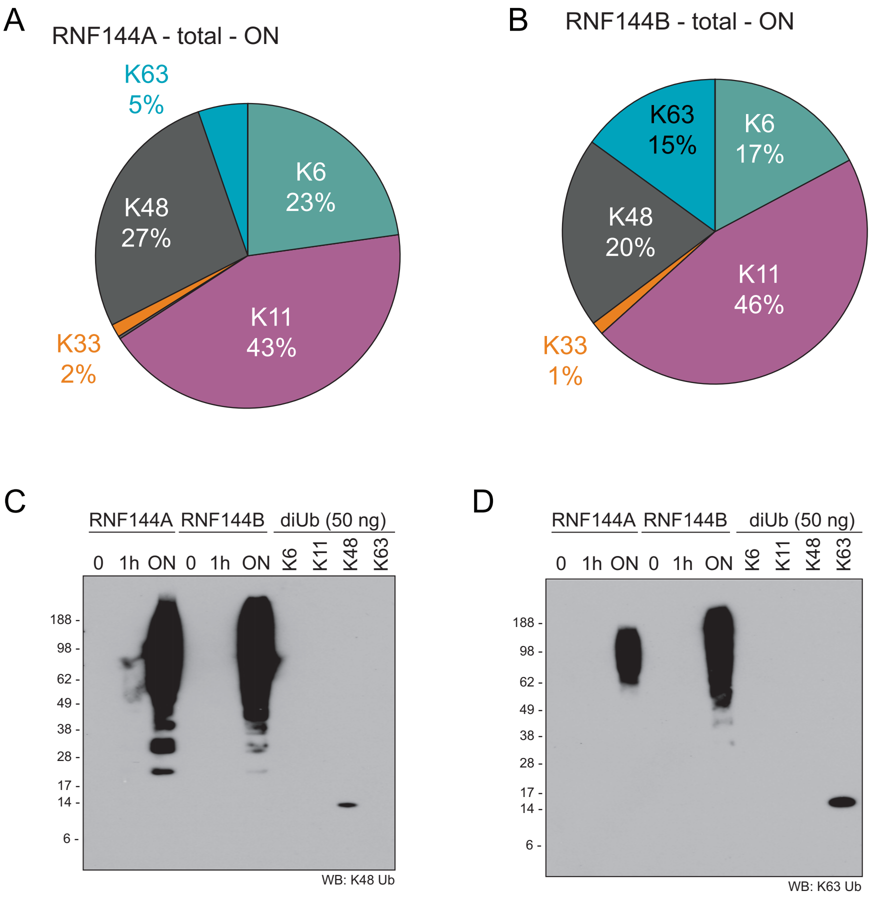
A
RNF144A - total - ON
K63
5%
K6
23%
K48
27%
K11
43%
K33
2%
B
RNF144B - total - ON
K63
15%
K6
17%
K48
20%
K11
46%
K33
1%
C
RNF144A
RNF144B
diUb (50 ng)
0
1h
ON
0
1h
ON
K6
K11
K48
K63
188 -
98 -
62 -
49 -
38 -
28 -
17 -
14 -
6 -
WB: K48 Ub
D
RNF144A
RNF144B
diUb (50 ng)
0
1h
ON
0
1h
ON
K6
K11
K48
K63
188 -
98 -
62 -
49 -
38 -
28 -
17 -
14 -
6 -
WB: K63 Ub

Figure S4 (related to Figure 4): ***In vitro*** **applications of Affimers**

A-B) AQUA-MS-derived Ub linkage composition for total assembly reaction of A) RNF144A and B) RNF144B after overnight assembly with UBE2L3 and Ub wt. **C-D)** *In vitro* assembly reaction of RNF144A and RNF144B with indicated amounts of recombinant diUb as in Figure 4D and probed with C) a K48-specific antibody or D) a K63-specific antibody. Longer chains are preferentially detected, probably due to avidity effects.

Figure S5

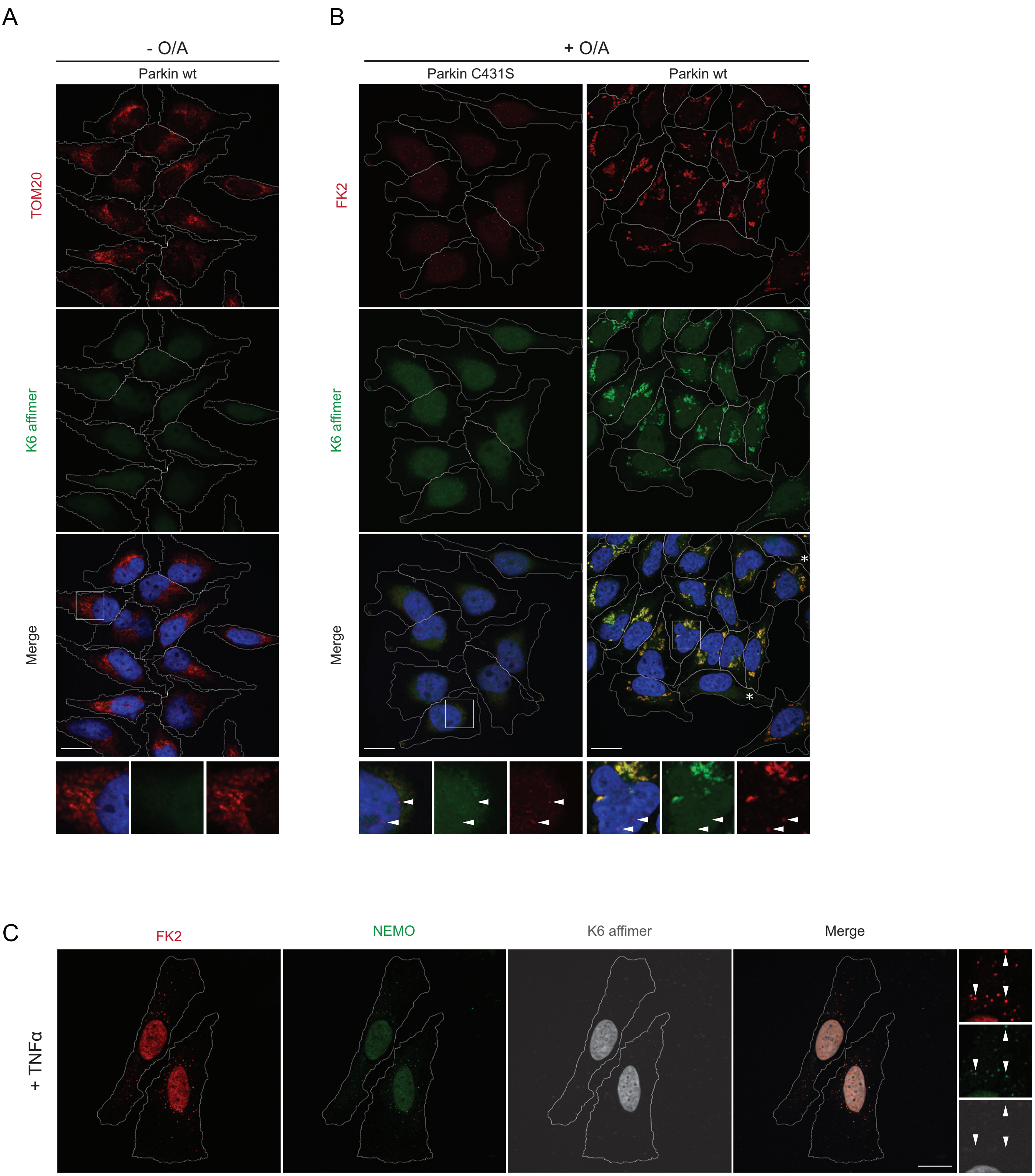
A
B
- O/A
+ O/A
Parkin wt
Parkin C431S
Parkin wt
TOM20
K6 affimer
Merge
FK2
K6 affimer
Merge
*
*
C
FK2
NEMO
K6 affimer
Merge
+ TNFα

Figure S5 (related to Figure 5): *In vivo* applications of Affimers

A) Confocal fluorescence microscopy images as in Figure 5C of untreated HeLa cells expressing wt Parkin stained with TOM20 (red), K6-affimer (green) and DAPI (blue). Scale bars correspond to 20 μm. **B)** As in A, but cells were treated with O/A and either wt Parkin or catalytically inactive (C431S) Parkin was expressed. Cells were stained with FK2 (red), K6-affimer (green) and DAPI (blue). Arrows indicate FK2-labelled ubiquitin conjugates that are not recognized by the K6-affimer. Not all cells seem to have ubiquitinated mitochondria (asterisks), either due to loss of Parkin expression, or because mitophagy was not induced. **C)** Confocal fluorescence microscopy images of cells that were treated with TNFα and stained with FK2 (red), NEMO (green), K6-affimer (grey) and DAPI (blue). NEMO-positive FK2 punctae are negative for the K6-affimer (white arrows) suggesting the affimer retains specificity in fluorescence staining of fixed cells.

Figure S6

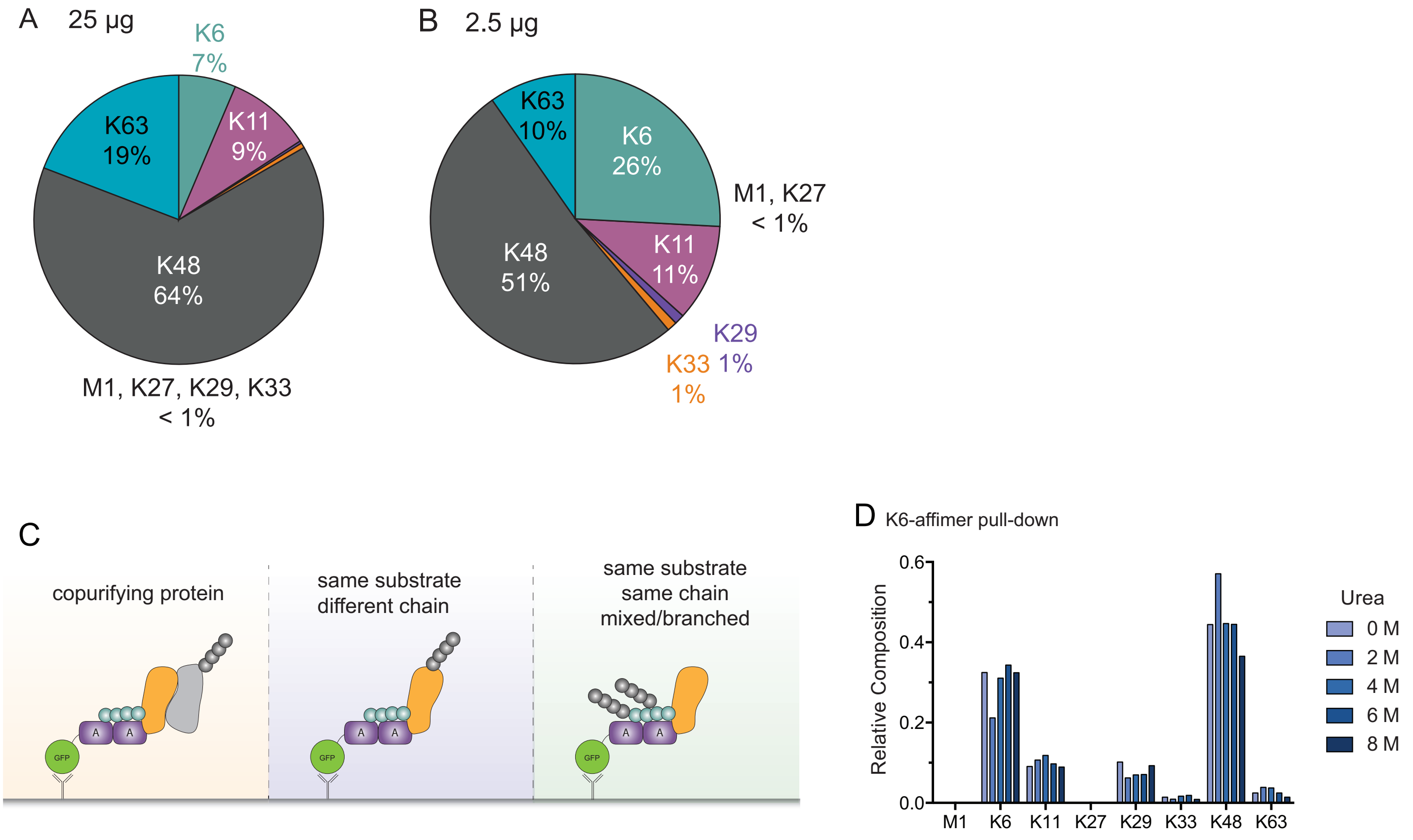
A 25 µg
K6
7%
K11
9%
K63
19%
K48
64%
M1, K27, K29, K33
< 1%
B 2.5 µg
K63
10%
K6
26%
M1, K27
< 1%
K48
51%
K11
11%
K29
1%
K33
1%
C
copurifying protein
same substrate
different chain
same substrate
same chain
mixed/branched
GFP
A
D K6-affimer pull-down
Relative Composition
0.0
0.2
0.4
0.6
M1
K6
K11
K27
K29
K33
K48
K63
Urea
0 M
2 M
4 M
6 M
8 M

Figure S6 (related to Figure 6): ***Enriching Ub linkages using affimers***
A-B) Ub linkage compositions from K6-affimer pull-downs performed with A) 25 µg and B) 2.5 µg of the K6-affimer. **C)** Schematic explaining the different scenarios likely explaining the origin of non K6-chains in K6-affimer pull-downs **D)** Relative Ub chain composition of K6-affimer pull-downs performed in the presence of the indicated concentrations of urea.

Figure S7

A

HUWE1 (aa)	PSMs		
	Rep 1	Rep 2	Rep 3
IPIPLMDYILNVMK (762-775)	-	1	-
FVESILSNNTTDDHCQEFVNQK (776-797)	2	-	-
MVNPTTVLESPHSLPAK (1209-1225)	-	5	3
WITPVLLLIDFYEK (1582-1595)	5	-	
IGEILIQGLTEDMVTVLIR (1742-1760)	2	-	-
SAATSGAGSTTSGVVSGSLGSR (1844-1865)	21	6	6
IVNQPSSLFGSK (2256-2267)	-	3	3
LLGPSAAADILQLSSSLPLQSR (2580-2601)	2	-	-
LLVGNDDVHIIAR (2606-2618)	3	-	-
AGSSTPGDAPPAVAEVQGR (2895-2903)	3	11	11
LLSLISIALPENK (3456-3468)	15	9	5

B

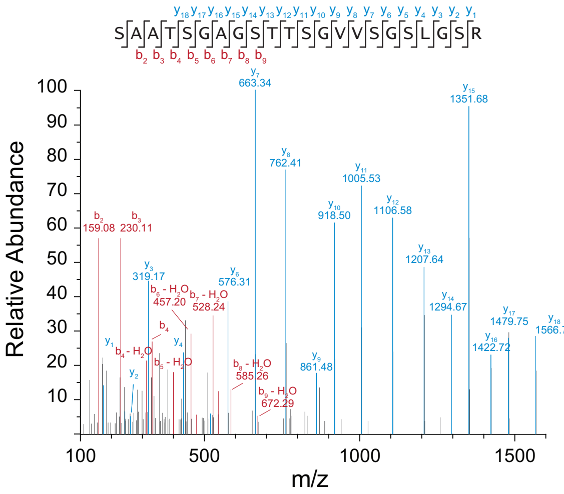

C

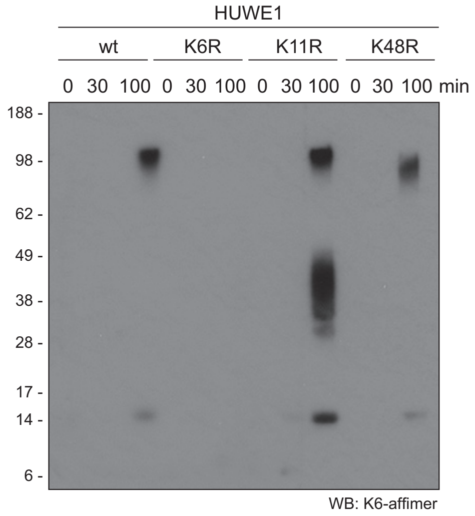

D

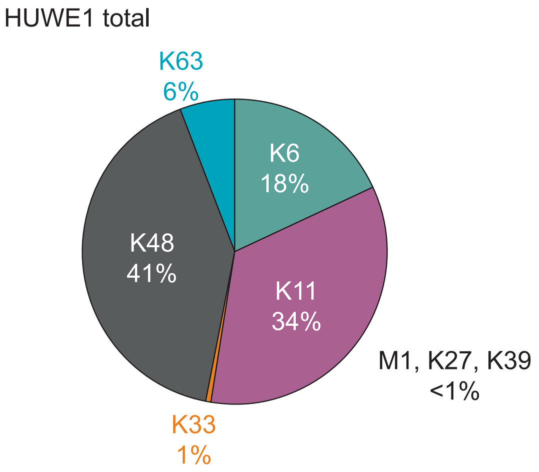

E

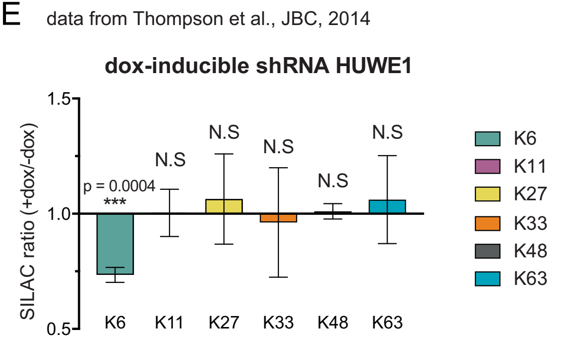

F

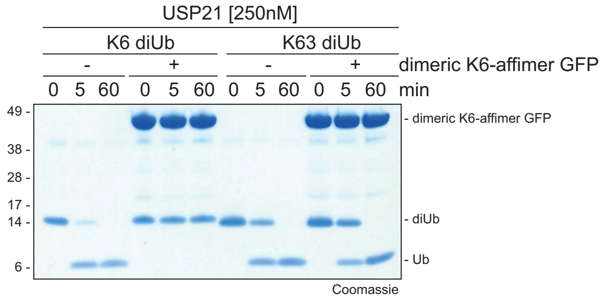

G

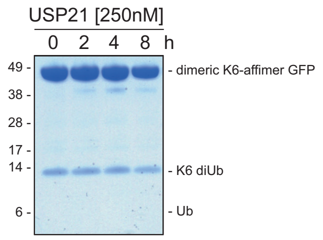

H

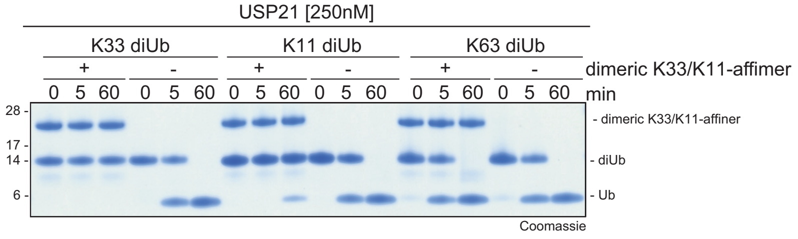

Figure S7 (related to Figure 7): HUWE1 assembles K6 chains *in vitro* and *in vivo*

A) Table summarizing the HUWE1 peptides identified in three replicate K6-affimer pull-down experiments with the corresponding number of PSMs. **B)** MS/MS fragmentation spectrum for one HUWE1 peptide (aa 1844-1865). **C)** Western blot with the K6-affimer of an assembly reaction with recombinant HUWE1 and different Ub mutants. **D)** Ub linkage composition of HUWE1-assembled Ub chains. **E)** SILAC ratios of induced vs. uninduced HUWE1 knockdown cell lines. Only singly modified peptides were analyzed. Data extracted and analyzed from Thompson et al., 2014. Error bars represent mean ± standard deviation. *** $p < 0.001$; N.S not significant as determined by a one sample, two-tailed *t* test. **F)** USP21 deubiquitinase assay in the presence or absence of GFP-tagged K6-affimer for K6 and K63 diUb. **G)** As in D, but with longer time points and only for K6 diUb. **H)** As in F but with K33/K11-affimer and for K33, K11 and K63 diUb.
